# PERK Is a Haploinsufficient Tumor Suppressor: Gene Dose Determines Tumor-Suppressive Versus Tumor Promoting Properties of PERK in Melanoma

**DOI:** 10.1371/journal.pgen.1006518

**Published:** 2016-12-15

**Authors:** Dariusz Pytel, Yan Gao, Katarzyna Mackiewicz, Yuliya V. Katlinskaya, Kirk A. Staschke, Maria C. G. Paredes, Akihiro Yoshida, Shuo Qie, Gao Zhang, Olga S. Chajewski, Lawrence Wu, Ireneusz Majsterek, Meenhard Herlyn, Serge Y. Fuchs, J. Alan Diehl

**Affiliations:** 1 Department of Biochemistry and Molecular Biology, Hollings Cancer Center, Medical University of South Carolina, Charleston, South Carolina, United States of America; 2 Department of Biomedical Sciences, School of Veterinary Medicine, University of Pennsylvania, Philadelphia, Pennsylvania, United States of America; 3 Oncology Discovery Research, Lilly Research Laboratories, Eli Lilly and Company, Lilly Corporate Center dc1104, Indianapolis, Indiana, United States of America; 4 Molecular and Cellular Oncogenesis Program, The Wistar Institute, Philadelphia, Pennsylvania, United States of America; 5 Department of Pathology and Laboratory Medicine, Medical University of South Carolina, Charleston, South Carolina, United States of America; 6 Department of Clinical Chemistry and Biochemistry, Medical University of Lodz, Lodz, Poland; Brigham and Women's Hospital, UNITED STATES

## Abstract

The unfolded protein response (UPR) regulates cell fate following exposure of cells to endoplasmic reticulum stresses. PERK, a UPR protein kinase, regulates protein synthesis and while linked with cell survival, exhibits activities associated with both tumor progression and tumor suppression. For example, while cells lacking PERK are sensitive to UPR-dependent cell death, acute activation of PERK triggers both apoptosis and cell cycle arrest, which would be expected to contribute tumor suppressive activity. We have evaluated these activities in the BRAF-dependent melanoma and provide evidence revealing a complex role for PERK in melanoma where a 50% reduction is permissive for Braf^V600E^-dependent transformation, while complete inhibition is tumor suppressive. Consistently, PERK mutants identified in human melanoma are hypomorphic with dominant inhibitory function. Strikingly, we demonstrate that small molecule PERK inhibitors exhibit single agent efficacy against Braf^V600E^-dependent tumors highlighting the clinical value of targeting PERK.

## Author Summary

PERK is critical for progression of specific cancers and has provided stimulus for the generation of small molecule PERK inhibitors. Paradoxically, the anti-proliferative and pro-death functions of PERK have potential tumor suppressive qualities. We demonstrate that PERK can function as either a tumor suppressor or a pro-adaptive tumor promoter and the nature of its function is determined by gene dose. Preclinical studies suggest a therapeutic threshold exists for PERK inhibitors.

## Introduction

Folding and maturation of secreted proteins occurs in the endoplasmic reticulum (ER). Cellular stresses that generate mis-folded proteins trigger a stress response termed the unfolded protein response pathway (UPR) [1–5]. Activation of the UPR is characterized by increased transcription of genes encoding ER molecular chaperones such as BiP/GRP78 and GRP94, protein disulfide isomerase, and CHOP (C/EBP homologous protein) [6–10]. Mammalian cells contain three ER transmembrane effectors of the UPR. Ire1 is composed of a luminal domain that senses stress, a single transmembrane domain, and a cytosolic tail that contains both a protein kinase domain and an Rnase domain [11, 12]. Ire1 regulates expression of numerous ER chaperones through activation of the X-box binding protein 1 (Xbp1) transcription factor [13]. Accumulation of Xbp1 is mediated by Ire1-dependent splicing that generates a shorter Xbp1 mRNA that is more efficiently translated [14, 15]. PERK, also an ER transmembrane protein kinase, is activated in a manner analogous to the Ire1 [16] and catalyzes serine 51 phosphorylation of eIF2α resulting in reduced protein synthesis [17–19]. The third signaling components are the transmembrane transcription factors ATF6α/β. While normally tethered to the ER, upon stress, ATF6 migrates to the trans-Golgi, where it is processed by S1P and S2P proteases to release the N-terminal DNA-binding transcription factor domain [20–22].

Physiologically, the UPR is an adaptive pathway. Through increased synthesis of chaperones, reduced protein synthesis and cell cycle arrest, cells have a window of opportunity to restore ER homeostasis prior to committing to apoptosis. Consistently, knockout of individual UPR signaling molecules, such as PERK or Ire1, severely compromises cell survival following stress [23–26]. When a cell is unable to alleviate the burden of mis-folded proteins, such as under conditions of chronic stress, the UPR triggers apoptosis [27–31]. Among the various pathways engaged, Perk-dependent activation of the pro-apoptotic CHOP transcription factor is the most heavily investigated [28–34]. The balance of pro-survival and pro-apoptotic signals following stress ultimately determines cell fate.

Although perturbations in protein folding in the ER can be achieved through the use of pharmacological agents that disrupt protein glycosylation (tunicamycin) or perturb calcium homeostasis (thapsigargin) [35–38], the rapid expansion of tumor cells results in a microenvironment wherein critical metabolic nutrients such as glucose, oxygen and growth factors become limiting resulting in UPR activation. Acute expression of oncogenes is also associated with UPR engagement [39–42]. Normal cells respond to chronic UPR activation via growth arrest and/or apoptosis thereby preventing cell expansion, while tumor cells typically bypass the anti-proliferative impact of UPR activation and instead depend upon the pro-adaptive signaling suggesting a potential point of therapeutic intervention. Indeed deletion of PERK can reduce tumor progression [42, 43]. Likewise, deletion of Xbp1, a transcription factor whose accumulation is dependent upon Ire1 activity, also reduces tumorigenesis [44]. Such results have stimulated attempts to generate small molecules that inhibit PERK or Ire1. Consequently, highly specific and potent inhibitors of the PERK enzyme have been developed [45–48].

While the UPR is considered important for tumor progression, there is potential for tumor suppressive activity given it antagonizes cyclin D1. With the advent of PERK specific inhibitors and an eye towards therapeutic utility, we have addressed the role of PERK in Braf^V600E^ driven melanoma and provide evidence for a dose-dependent function of PERK in melanoma genesis.

## Results

### Braf^V600E/+^; Perk+/-deficient mice develop melanoma

**Fig 1:**
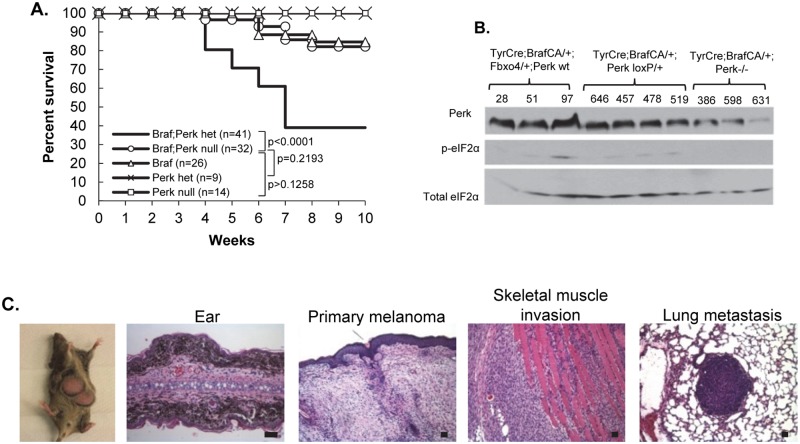
Deletion of one Perk allele cooperates with Braf^V600E^ to drive metastatic melanoma. **A)** Kaplan-Mayer survival curve of TyrCre+/-;Braf^V600ECA/+^;Perk+/- mice treated with 4-HT. **B)** Western blot of melanoma skin lysates from TyrCre+/-;Braf^V600ECA/+^;Fbxo4;Perk +/+ or TyrCre+/-;Braf^V600ECA/+^;Perk+/- or Perk-/- mice (blot anti-Perk, p-eIF2α, eIF2α). **C)** Melanoma from TyrCre+/-;Braf^V600ECA/+^;Perk+/- mice analyzed by H&E. Scale bars = 50μm.

**Fig 2:**
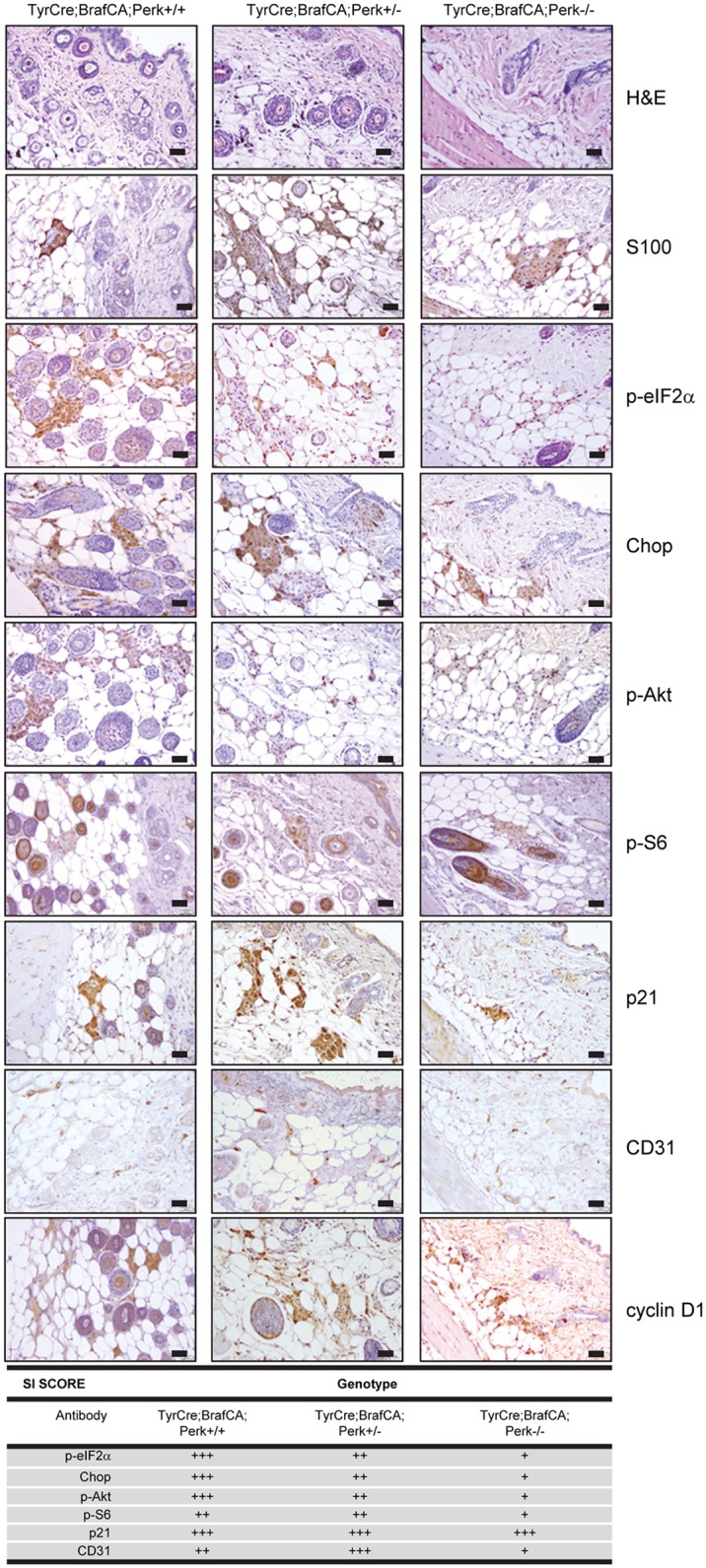
Mono-allelic Perk deletion reduces pro-apoptotic signals and maintains pro-survival signaling. H&E and IHC of premalignant skin from TyrCreCA/+;Braf^V600ECA/+^, Perk +/+ or Perk+/- and Perk-/- mice for the indicated targets and quantification of staining/staining index (SI) of IHC; scale bars = 50μm.

Perk harbors anti-proliferative activity [49] in addition to cell survival activities, suggesting a potential for tumor suppressive properties. We ascertained the impact of deletion of one versus two alleles of Perk in melanocytes harboring activated Braf^V600E^. We utilized a conditional allele of Perk to circumvent issues of pancreatic atrophy that occurs in a global Perk knockout [50–52]. Previous work with the mice wherein Braf^V600E^ expression alone is induced in melanocytes revealed induction of cellular senescence rather than tumor development [53]. Bypass of Braf^V600E^-dependent senescence has only been observed in mice wherein a second tumor suppressor such as p16^Ink4A^ [54–57], PTEN [53], or Fbxo4 has been deleted [58]. Remarkably, Braf^V600ECA/+^;Perk+/- mice developed melanoma with high penetrance within 4–6 weeks which rapidly disseminated to peripheral tissue (Fig 1A–1C). Immunohistochemistry (IHC) for S100 confirmed melanocytic origin melanocytes (Fig 2).

To address underlying mechanisms, we analyzed “pre-malignant skin” from TyrCre; Braf^CA/+^;Perk +/+ or Perk+/- or Perk -/- mice. IHC revealed reduced accumulation of, p-Akt and pS6 in Perk+/- skin relative to wt, but higher than Perk-/- consistent with dosage dependence (Fig 2). Likewise, consistent with the presence of one wild type Perk allele, modestly elevated p-eIF2α and CHOP was observed relative to Perk-/- tissue. Braf^V600ECA/+^;Perk+/- tissue exhibited the highest level of staining CD31 staining, with Braf^V600ECA/+^;Perk+/+ having intermediate levels and Braf^V600ECA/+^;Perk-/- exhibiting the lowest level consistent with previous work [10, 59]. These results demonstrate that deletion of one allele of Perk reduces p-eIF2α, pro-apoptotic CHOP yet maintains or even increases vascularity, as determined by CD31 staining.

### Deletion of one PERK allele reduces Braf^V600E^-induced senescence and drives cyclin D1-dependent melanoma

**Fig 3:**
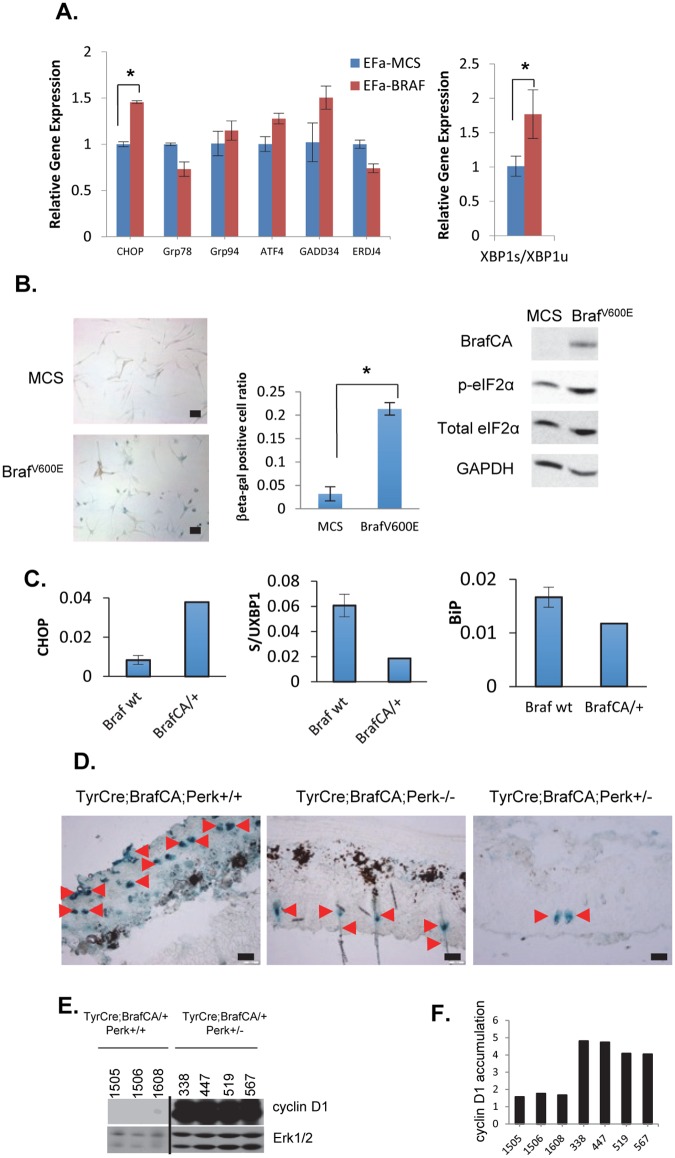
Braf^V600E^ induces ER stress and reduced PERK gene dosage attenuates Braf^V600E^-dependent senescence in premalignant skin. **A)** Braf^V600E^-dependent regulation of ER stress genes (CHOP, Grp78, Grp94, ATF4, GADD34, ERDJ4, XBP1) in primary melanocytes (QPCR; *p<0.05; p-values analyzed by two-tailed Student t test) **B)** β-galactosidase staining of primary Braf^WT^ vs Braf^V600E^ melanocytes. (Scale bars 50μm; *p<0.05; p-values analyzed by two-tailed Student t test); western analysis of cell lysates using indicated antibodies. **C)** UPR gene expression assay (QPCR) for Chop, Xbp1, Bip in premalignant skin from Braf^WT^ or Braf^V600E^ mice. **D)** β-galactosidase assay in premalignant skin isolated from TyrCreCA/+; Braf^V600ECA/+^; Perk+/+ or Perk+/- or Perk-/- mice. Arrowheads indicate positive staining. Scale bars = 50μm. **E)** Detection of cyclin D1 in the tissues indicated by immunoblot. Total Erk1/2 is provided as a loading control. The line denotes excision of irrelevant lanes. **F)** Quantification of E. Cyclin D1 protein levels expressed as a ratio relative with total ERK.

The observation that PERK+/- melanocytes are permissive for Braf^V600E^-dependent transformation implies that acute activation of Braf^V600E^ triggers PERK activity and PERK tumor suppression. To assess this hypothesis, primary human melanocytes were infected with retrovirus encoding Braf^V600E^. Expression of mutant Braf triggered increased p-eIF2α and elevated CHOP (Fig 3A and 3B). Conversely chaperone expression was not increased suggesting the absence or weak activation of ATF6 (Fig 3A). Braf^V600E^ expression was associated with increased SAβ-galactosidase consistent oncogene induced senescence (Fig 3B). Armed with evidence for Braf^V600E^-dependent activation of Perk in vitro, we assessed Braf^V600E^-dependent activation of Perk in vivo. Following activation of Braf^V600E^ expression specifically in melanocytes with topical application of 4-OHT, we noted increased expression of Chop; however, no increase in chaperone expression was observed and Xbp1 splicing was reduced suggesting that Braf^V600E^ selectively induces Perk in vivo (Fig 3C). To assess oncogene induced senescence, we measure SA-βGal in the skin of mice harboring Braf^V600E^ in Perk+/+, +/- and -/- backgrounds. Here we noted reduced SA-βGal staining specifically in Perk+/- relative to +/+ tissue demonstrating that deletion of one Perk allele permitted bypass of Braf^V600E^-induced senescence (Fig 3D; quantification, S1 Fig). We also noted significant overexpression of cyclin D1 in Braf^V600ECA/+^/Perk+/- relative to Braf^V600ECA/+^/Perk+/+ (Fig 3E and 3F). Consistent with Perk functioning as an antagonist of cyclin D1 protein synthesis [60].

**Fig 4:**
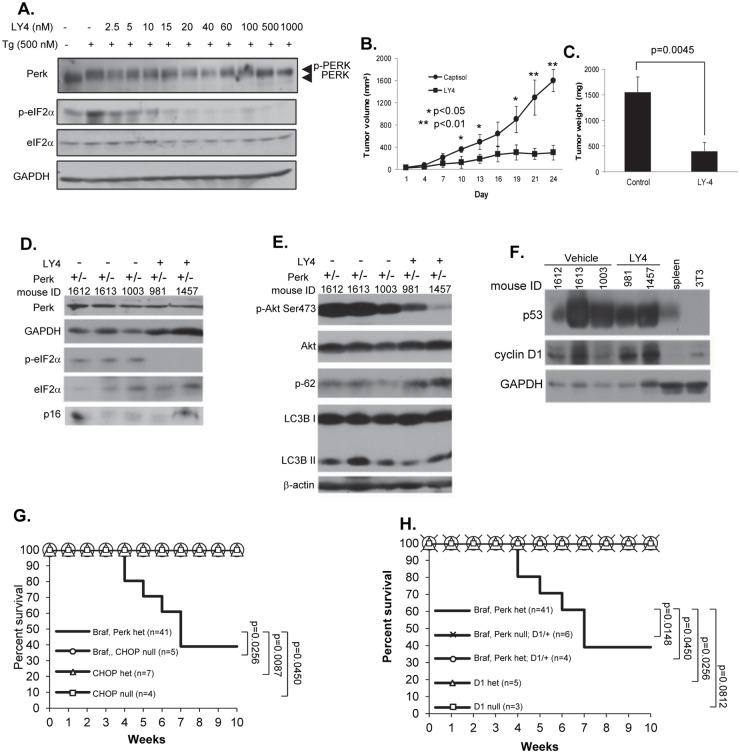
Braf^V600ECA/+^;Perk+/- tumors are dependent upon the remaining Perk allele. **A)** LY4 PERK inhibitor inhibits stress-dependent PERK signaling in cultured melanoma cells. **B-C)** Measurement of melanoma tumor volume B) and tumor weight C) in TyrCre+/-;Braf^V600ECA/+^;Perk +/- mice treated with LY-4; p-values analyzed by two-tailed Student t test. **D-F)** Western blot of melanoma skin lysates obtained from TyrCre+/-;Braf^V600ECA/+^;Perk +/- mice treated with PERK inhibitor LY-4. **G)** Kaplan-Mayer survival curve of TyrCre+/-;Braf^V600ECA/+^;Chop+/- or TyrCre+/-;Braf^V600ECA/+^;Chop-/- mice expressed relative to control genotypes. **H)** Kaplan-Mayer survival curve for Braf^V600ECA/+^;D1+/-;Perk+/- expressed relative to control genotypes.

**Fig 5:**
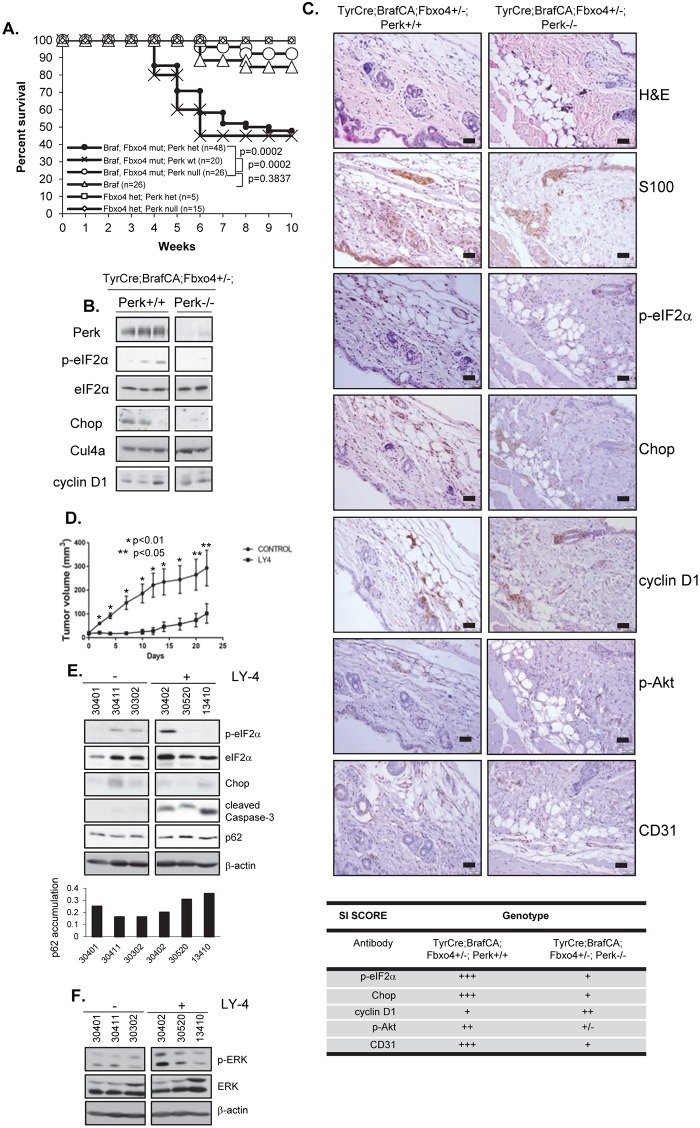
Bi-allelic Perk deletion abolishes Braf^V600ECA/+^;Fbxo4+/- melanoma initiation, but Perk inhibition cannot recede tumor progression. **A)** Kaplan-Mayer survival curve of Braf^V600ECA/+^, Fbxo4 +/- and Perk +/+, +/- or -/- mice treated with 4-Hydroxytamoxifen (4-HT). **B)** Western blot of melanoma lysates from TyrCreCA/+; Braf^V600ECA/+^; Fbxo4 +/-; Perk +/+, Perk+/- or Perk-/- mice **C)** IHC analysis of skin from TyrCre+/-;Braf^V600ECA/+^;Fbxo4 +/-; Perk+/+ or Perk -/- mice; H&E or IHC with antibodies for S100, p-eIF2α, Chop, cyclin D1, p-Akt, CD31 and quantification of staining/staining index (SI) of IHC. Scale bars = 50μm. **D)** Tumor volume in TyrCre+/-;Braf^V600ECA/+^;Pten-/- mice treated with LY-4; p-values analyzed by two-tailed Student t test. **E-F)** Western blot of melanoma skin lysates from TyrCre+/-; Braf^V600ECA/+^;Pten-/- mice treated with LY-4.

The susceptibility of Perk+/- but not Perk-/- mice to Braf^V600E^-dependent melanoma genesis, along with the retention of 50% Perk protein expression in all tumors examined (Fig 1) suggested the intriguing possibility that the remaining Perk allele was necessary for malignant progression. We addressed whether the remaining Perk allele was necessary for tumor progression by treating tumor-bearing mice with LY-4. LY-4 is a PERK specific inhibitor with a 2nM IC50 and little activity towards other eIF2α kinases (S1 Table). Kinome and Treespot analysis demonstrates the selectivity of LY-4 for PERK relative to > 400 additional kinases (S2A and S2B Fig; S2 Table). After confirming that LY-4 inhibits PERK activity in cultured melanoma cells (Fig 4A), mice were exposed to tamoxifen to induce BRAF^V600E^ and delete a single Perk allele; LY-4 treatment was initiated when tumors were 2-3mm^3^. LY-4 treatment reduced tumor growth by nearly 90% (Fig 4B and 4C). Treatment reduced p-eIF2α (Fig 4D), p-Akt, [61]; LY-4 treatment also elevated p62 and reduced LC3BII (Fig 4D–4F) consistent with reduced autophagy. LY-4 also reduced phospho-H3, Ki67 and CD31, while increasing TUNEL positivity demonstrating reduced proliferation and increased apoptosis (S3A Fig) as mechanisms contributing to LY-4-dependent tumor inhibition. No pancreatic toxicity was noted in LY-4 treated animals (S3B and S3D Fig). Finally, LY-4 did not inhibit MAPK activation (Fig 5F; S3C Fig) demonstrating its impact on tumor growth does not reflect inhibition of downstream Braf^V600E^ targets.

The observation that Perk+/- was permissive while Perk-/- was resistant to Braf^V600E^ melanoma, suggested a model where tumors remained dependent upon Perk for its pro-survival activity, but that reduced Perk dosage permitted senescence bypass, through either lack of apoptosis (e.g. reduced CHOP induction) or cyclin D1 induction reflecting reduced inhibition of translation under Perk deficiency. To test this, we 1) reduced expression of pro-apoptotic Chop or 2) reduced cyclin D1 levels. If reduced Chop was a key progression, Chop+/- or -/- mice should be susceptible to Braf^V600E^-melanoma. However, deletion of Chop in Braf^V600ECA/+^ mice did not permit melanoma development (Fig 4G). To evaluate the role of cyclin D1 overexpression, in Braf^V600E^/Perk+/- tissue, we deleted one allele of CCND1 and deletion completely abrogated melanoma genesis (Fig 4H). Overexpression of cyclin D1 drives development of lymphomas by triggering DNA damage, which in turn activates p53 [62, 63]; as such, tumor progression selects either apoptosis or p53 loss [62, 64–66]. Consistent with cyclin D1 overexpression contributing to melanoma in the Perk+/- background, p53 was overexpressed suggesting stabilizing mutations (Fig 4F). DNA sequencing revealed p53 mutations in 7 of 7 tumors with mutations apparent throughout the DNA-binding domain (S3 Table).

### Melanoma genesis in Braf^V600E^;Fbxo4 mutant mice are dependent upon one functional Perk allele

The results presented above reveal that Perk+/- melanocytes are permissive for Braf^V600E^-melanomagenesis while, Perk-/- are not. The capacity of LY-4 to inhibit progression of Braf^V600E^;Perk+/- melanomas, implies an “addiction” to the remaining Perk allele suggesting a potential therapeutic threshold for Perk inhibition. To address Perk function in a mouse model of metastatic melanoma [58], we generated Tyr-Cre/Braf^V600ECA/+^/Fbxo4^mt^/Perk^f/f^ or Tyr-Cre/Braf^V600ECA/+^/Fbxo4^mt^/Perk^f/+^ permitting inducible activation of Braf^V600E^ and deletion of one or two alleles of *Perk* in melanocytes upon application of 4-OHT. We have previously demonstrated that inactivation of Fbxo4 in the Braf^V600ECA/+^ background triggers cyclin D1-dependent, metastatic melanoma [58]. Deletion of both Perk alleles effectively inhibited Braf^V600E^-dependent melanoma in the setting of Fbxo4-deficiency, while deletion of one allele of *Perk* (Perk+/-) was not sufficient to either inhibit or accelerate melanoma genesis (Fig 5A; S4A Fig). The absence of tumor inhibition is consistent with data in Fig 1 demonstrating the permissiveness of Perk+/- melanocytes to Braf^V600E^. The fact that we do not observe decreased latency in the Fbxo4mt/Perk+/- background is consistent with both Fbxo4 and Perk signaling converging on the inhibition of cyclin D1.

To address mechanism, we assessed Perk activity and downstream readouts in premalignant skin. Perk deletion reduced eIF2α phosphorylation and Chop expression in premalignant skin as detected by western analysis (Fig 5B). The variability in signal likely reflects the fact that Perk is only deleted in melanocytes and we are analyzing whole skin. We next assessed various markers by IHC. We first stained sections with S100 to identify melanocytes and subsequent sections with the antibodies indicated. Deletion of Perk was associated with increased cyclin D1 specifically in pre-malignant melanocytes (Fig 5C) consistent with Perk-antagonizing cyclin D1 translation [49, 67, 68]. IHC also revealed decreased p-Akt and CD31 consistent with Perk-dependent regulation of both Akt signaling [61, 69–71] and angiogenesis [72–74] (Fig 5C).

We next utilized Braf^V600E^;Pten-/- mice, an independent melanoma model, to determine whether Perk was required for tumor progression [75, 76]. Mice were exposed to 4-OHT to induce BRAF^V600E^ and delete Pten; LY-4 treatment was initiated when tumors were 2-3mm^3^. LY-4 inhibited melanoma progression (Fig 5D, S4E Fig) and this outcome was accompanied by reduced eIF2α phosphorylation and CHOP accumulation suggesting on-target effects of this drug (Fig 5E). LY-4 treatment also led to accumulation of p62 suggesting reduced autophagy, and elevated cleaved caspase 3 indicative of increased rate of apoptosis (Fig 5E). IHC confirmed that LY-4 treatment resulted in decreased p-eIF2α, Chop, and p-H3 (S4B Fig). Reduced CD31 staining was also noted (S4B Fig), consistent with previous work linking Perk signaling with tumor angiogenesis [72–74]. IHC also revealed increased TUNEL positive tumor cells (S4B Fig). We also monitored blood glucose levels in LY-4 treated mice, given previous evidence that Perk inhibition caused pancreatic toxicity (45). Importantly, blood glucose levels remained stable (blood glucose level was lower than 200 mg/dL), with no evidence of pancreatic damage during the course of treatment (S4C and S4D Fig).

### Human melanoma-derived cells are dependent on functional PERK

**Fig 6:**
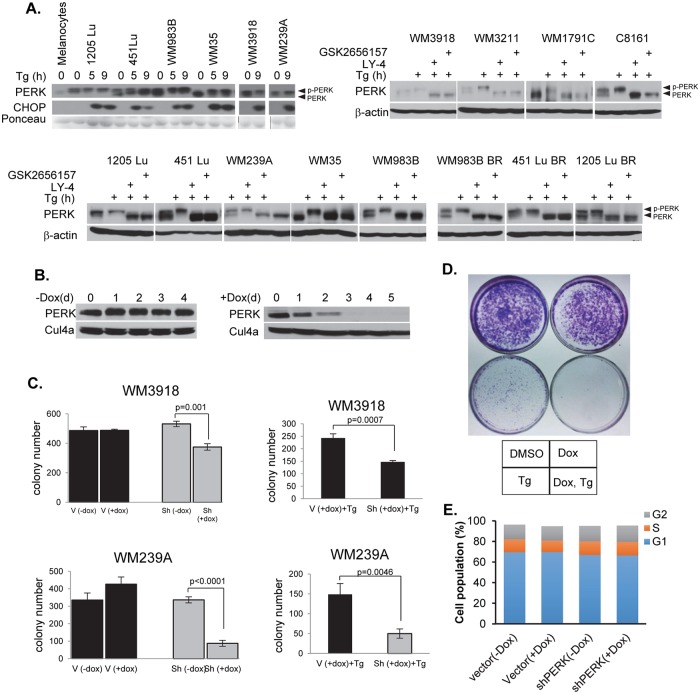
PERK is active in human melanoma cell lines and is required for melanoma genesis. **A)** PERK is functional human melanoma cell lines. **B)** Dox-dependent PERK knockdown in WM3918 cells. **C)** WM3918 and WM239A growth in soft agar with or without PERK knockdown, +/- thapsigargin (Tg); V-vector; doxycycline (Dox); shPERK (soft agar); p-values analyzed by two-tailed Student t test **D)** Clonogenic survival assay of PERK knockdown WM3918 cells treated with thapsigargin (Tg); doxycycline. **E)** Cell cycle profile of PERK knockdown WM3918 cells.

If melanoma progression-depends upon the retention of functional PERK, it stands to reason that human melanoma-derived cell lines will maintain and depend upon PERK activity. To address the contribution of PERK to melanoma, we determined whether PERK was functional in melanoma cell lines. We utilized melanoma cells lines lacking detectable mutations in PERK, but expressing the following Braf alleles: BrafWT or Braf^V600E/D^ (1205 Lu, 451LU, WM983B, WM35, WM3918, WM239A, WM3211, WM1791C, C8161 (http://www.wistar.org/lab/meenhard-herlyn-dvm-dsc/page/melanoma-cell-lines-0). PERK expression was detected and was functional in all cell lines assessed (Fig 6A). To assess PERK contributions to melanoma cell survival following ER stress, we established two independent melanoma cell lines (WM3918, WM239A) expressing a previously validated, tetracycline-inducible shRNA directed against human PERK [43, 77]. PERK expression was undetectable 3-days post-doxycycline in shRNA-harboring cells (Fig 6B). To assess whether PERK function is important for melanoma cell growth and survival, we took advantage of previous work revealing a role for PERK in promoting survival following cell detachment from solid matrix [78, 79]. Consistently, PERK knockdown in either WM3918 or WM239A reduced colony formation in soft agar (Fig 6C; left graphs). Addition of thapsigargin further reduced anchorage-independent growth (right graphs). PERK function also increased cell survival in clonogenic survival assays (Fig 6D). In contrast, PERK knockdown cells grew well on plastic under normal growth conditions (Fig 6D top; Fig 6E). As an independent method for assessing PERK contribution to melanoma cell survival thereby ensuring that the impact of shRNA was PERK-dependent, we utilized a previously characterized small molecule inhibitor of PERK, GSK2656157 [45–47] or LY-4. GSK2656157 or LY-4 treatment inhibited PERK activity (as judged by reduced auto-phosphorylation) and suppressed melanoma cell survival under ER-stress (S5A and S5B Fig). These data demonstrate that retention of functional PERK is critical for melanoma cell survival.

### PERK mutants found in human melanomas are hypomorphic and capable of promoting tumorigenic phenotype

**Fig 7:**
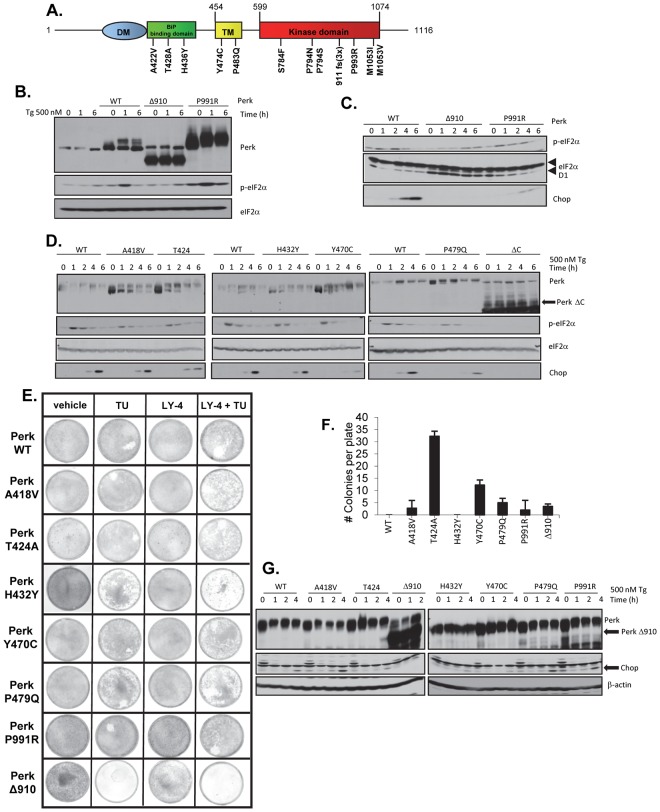
Characterization of a tumor-derived mutation of the PERK kinase. **A)** Depection of melanoma-derived PERK mutations. **B-D)** Western analysis of MEF lysates expressing murine versions of Perk alleles tested under ER-stress conditions. **E)** Clonogenic survival assay of Perk-/- fibroblasts reconstituted with Perk alleles (indicated on left) and treated with with chemicals indicated at the top. Cells stained with Geimsa after 14 days post-treatmen. **F)** Quantification of colonies that formed in soft agar for Perk-/- fibroblasts reconsituted with the indicated Perk mutants. **G)** Tumor-derived Perk alleles exhibit dominant negative activity.

The susceptibility of the PERK+/- genotype to melanoma genesis suggests a potential for inactivation of PERK in human melanoma. We searched the human cancer genome atlas and identified mutations throughout PERK coding exons at a frequency of ~7% (Fig 7A). To assess the functional consequence of these mutations to PERK function, we generated analogous alleles in murine Perk (A418V, T424A, H432Y, Y470C, P479Q, P991R, Δ910-analogous to human 911fs which deletes AA910-1116) and reconstituted Perk-/- MEFs by retroviral transduction. Importantly, all mutants remained localized to ER structures analogous to wild type Perk (S6B Fig). Nevertheless, all mutants exhibited reduced activities with PerkΔ910 exhibiting the least activity as determined by p-eIF2α, Chop induction and cyclin D1 repression (Fig 7B and 7D). Consistent with all mutations compromising Perk function, Perk-/- MEFs reconstituted with melanoma-derived Perk mutants exhibited increased sensitivity to ER stress as determined by clonogenic survival assay (Fig 7E). Importantly, co-administration of LY-4 with tunicamycin further reduced cell survival demonstrating that mutant Perk allele activity still contributed to survival following ER stress and that all alleles remained LY-4 sensitive (Fig 7E). Given that reduced Perk function cooperated with BRAF in vivo, we expected that hypomorphic Perk might increase spontaneous cell transformation. Consistently, cells expressing mutant Perk formed foci and grew in soft agar, both surrogates of cell transformation (Fig 7F; S6A Fig).

Finally, because Perk mutations will occur in the context of one wild type Perk allele, we considered the potential of these tumor-derived Perk mutants to exhibit dominant negative activity relative to endogenous Perk. To address this, we transduced NIH3T3 cells, which retain wild type Perk, with retrovirus encoding selected mutant Perk alleles (Fig 7G). Following transduction, cells were exposed to thapsigargin and we subsequently measured Chop expression as a read out of Perk function. Consistent with dominant negative activity, we noted reduced Chop expression in cells expressing most Perk mutants. This data reveals that hypomorphic alleles of Perk exhibit dominant inhibitor activity with respect to endogenous Perk and suggests physiological relevance of Perk mutants, in melanoma development.

## Discussion

While PERK has pro-survival and thus pro-tumorigenic activities, it also triggers pro-apoptotic signals and opposes cell division via inhibition of cyclin D1 translation. This latter PERK attribute begs the question of whether under certain conditions, or in specific tissues, PERK might function as a tumor suppressor. Supporting potential tumor suppressive function for the UPR, HRAS-dependent transformation of primary human melanocytes was potentiated by genetic inhibition of PERK, Ire1 and ATF4 [40]. To directly assess the contribution of Perk to melanoma genesis in vivo, Perk was deleted in a mouse melanocytes coordinately with activation of Braf^V600E^ [58]. Deletion of single Perk allele resulted in melanocytes permissive to overt transformation through expression of Braf^V600E^ alone. Typically, Braf^V600E^-dependent melanoma can be achieved by deletion or inactivation of known tumor suppressors, such as PTEN [53, 80], p16^Ink4A^ [81–83], Fbxo4 [58]. In the absence of inactivating mutations in one of these genes, Braf^V600E^ expression is associated with permanent growth arrest or senescence. Mono-allelic deletion of Perk thus represents a previously unappreciated mechanism for bypass of Braf^V600E^-dependent senescence. In addition, it is the only example wherein deletion of one versus two alleles results in diametrically opposing results with regard to tumor suppression versus tumor progression.

The basis of Perk haploinsufficiency likely reflects dose-dependent signaling duration and/or intensity. Accordingly, loss of one allele reduces pro-apoptotic signals (CHOP expression reduced), increases expression of a pro-oncogenic protein (cyclin D1) and maintains sufficient pro-survival signals through the remaining allele. Additionally, we noted that excision of one Perk allele suppressed Braf^V600E^-induced senescence. This likely reflects dysregulation of cyclin D1, given previous work that associates cyclin D1/CDK4 function with senescence [84, 85].

Importantly, Braf^V600E^/Perk+/- melanomas are dependent upon the remaining Perk allele. There have been sporadic reports implicating potential tumor suppressor like functions for Perk. Acute ablation of Perk in mammary epithelium increased tumor formation due to the accumulation of genomic instability [43]. Anti-proliferative activity of Perk was also attributed to differential impacts on mammary tumorigenesis [79].

In contrast to mono-allelic deletion, excision of both Perk alleles did not cooperate with Braf^V600E^ demonstrating that retention of one functional allele of Perk is required for tumor progression. The demonstration that Braf^V600E^/Perk+/- melanomas are dependent upon the remaining Perk allele supports this conclusion. These results reveal an unanticipated role for Perk in melanoma initiation, given previous work arguing that Perk is not required for tumor initiation [42, 43]. Bi-allelic Perk deletion did not impact oncogene-induced senescence or Erk1/2 phosphorylation demonstrating Perk is not required for Braf^V600E^ signaling. This data reveals that Perk is required for the establishment of Braf^V600E^ melanoma, but does not address whether PERK is a therapeutic target. Strikingly, treatment of mice with established Braf^V600E^/PTEN-dependent tumors triggered significant inhibition of tumor growth providing support for Perk as a bona fide therapeutic target. LY-4 treatment was associated with increased apoptosis, reduced markers of angiogenesis and decreased proliferation. Importantly, LY-4 treatment inhibited eIF2α phosphorylation and CHOP induction demonstrating on target effects of this agent.

While a reduction in CHOP expression may also facilitate tumor progression by limiting cell death, it is not the primary driver susceptibility, as a Chop deletion in the BrafV600E background does not permit melanoma genesis. In contrast, cyclin D1 overexpression is a key tumorigenesis-driving event. Dysregulation of the cyclin D1/CDK4 pathway occurs in a majority of melanomas and increased cyclin D1/CDK4 activity (e.g. loss of p16^Ink4A^ or Fbxo4) cooperates with Braf^V600E^ to drive melanoma. Second, reducing cyclin D1 expression through by deletion of a single allele inhibits melanoma genesis. Third, we have previously shown that cyclin D1-driven tumors are specifically opposed by p53 [63] and p53 is subject to mutations within its DNA binding domain in Perk+/- tumors while p16^Ink4a^ is still expressed.

If Perk heterozygosity were relevant to Braf^V600E^-dependent human melanoma, one would predict the occurrence of inactivating mutations in PERK in human melanoma. Consistently PERK mutations occur at a frequency of approximately 7%. Initial characterization of these mutants revealed reduced PERK activity. Cells expressing these mutants fail to effectively repress cyclin D1, exhibit reduced CHOP expression and are prone to spontaneous transformation, analogous to cyclin D1 overexpressing cell lines.

Collectively, our data reveals a complex role for Perk in melanoma genesis. While Perk pro-survival activity is necessary for melanoma genesis and melanoma progression, its ability to antagonize cell division through cyclin D1 supports a model wherein Perk regulation of cell fate is a delicate balance wherein less is not necessarily better. Importantly, LY-4 exhibited clear therapeutic potential for Braf^V600E^-dependent melanoma. Since Perk mutant tumors are dependent upon the remaining Perk allele, Perk status is unlikely to feature into patient response. An open question remains as to whether Perk tumor suppressive function is tissue specific. While previous work revealed no evidence for such in mammary tissue (43), additional analysis is required to address this issue.

## Methods

### Ethics statement

All animal use and experiments were approved by The Medical University of South Carolina Office of Compliance and Institutional Animal Care and Use Committee (IACUC) (approved animal use protocol #AR3340).

### Animal husbandry

All animals and experiments were carried out in compliance with Institutional Animal Care and Use Committee guideline of Medical University of South Carolina. Animals were obtained from the followings: TyrCreER, Braf^CA/+^, Fbxo4^+/-^ [58]; Perk^l/l^ [51]CyclinD1^+/-^ (from Jackson Laboratory); Chop^-/-^ (from Jackson Laboratory, stock #: 005530). Appropriate intercrosses were established to generate TyrCre;Braf^V600ECA/+^;Fbxo4+/- or -/-;Perk^+/+^, TyrCre;Braf^CA/+^;Fbxo4+/-or-/-; Perk^l/+^, TyrCre;Braf^CA/+^;Perk^l/+^, TyrCre;Braf^V600ECA/+^;Perk^l/+^;D1^+/-^, TyrCre;Braf^V600ECA/+^;Chop^+/-^or^-/-^ mice and controls.

For LY-4 treatment studies, tumor volume was measured twice per week and calculated with the following formula: volume = (length × width × width)/2. LY-4 treatment was initiated when a tumor reached ~3mm^3^ (formulated in 20% Captisol in 25mM of NaH_2_PO_4_ buffer, pH 2.0) by oral gavage twice per day. Blood glucose was monitored every five days using a Freestyle meter (TheraSense, Inc.) during LY-4 treatment.

Animal experiments were conducted in accordance with IACUC protocols and University Laboratory Animal Research (ULAR) guidelines. 4-Hydroxytamoxifen (4-OHT) was freshly prepared in dimethyl sulfoxide (DMSO) (5mM) and applied topically for three consecutive days to postnatal day 2 pups.

### Real-time PCR

The mouse skin tissue and human primary melanocytes were homogenized in Trizol (Invitrogen) and total RNA was extracted with chloroform. The cDNA was synthesized by using MMLV reverse transcriptase III and random primers (Invitrogen) following the manufacturer’s protocol. QPCR assay was prepared by using SyBr PCR mix (Applied Biosystems) and amplified using ABI Prism 7000 Sequence Detection System (Applied Biosystems) with the following primers: Chop (F: 5’-CCAACAGAGGTCACACGCAC-3’; R: 5’-TGACTGGAATCTGGAGAGCGA-3’), Uxbp1 (F: 5’-CACCTTCTTGCCTGCTGGAC-3’; R: 5’-GGGAGCCCTCATATCCACAGT-3’), Sxbp1 (F: 5’-GAGTCCGCAGCAGGTG-3’; R: 5’-GTGTCAGAGTCCATGGGA-3’), Bip (F: 5’-ACCCTTACTCGGGCCAAATT-3’; R: 5’-AGAGCGGAACAGGTCCATGT-3’), Gapdh (F: 5’-GGAGCGAGACCCCACTAACA-3’; R: 5’-ACATACTCAGCACCGGCCTC-3’). Infected skin melanocytes were lysed directly with TRIzol reagent (Sigma) followed by the isolation of total RNA according the user’s instructions. One μg total RNA was reverse transcribed using a Maxima First Strand cDNA Synthesis Kit for qRT-PCR (Thermo Fisher). Fast SYBR. Green Master Mix (life Technologies) was used with cDNA template and primers to evaluate the expression of target genes and GAPDH. Primers used were purchased from Integrated DNA Technologies. Amplifications were performed using an Applied Biosystems. 7500 Real-Time PCR System (Life Technologies). All experiments were performed in triplicate. Expression ratios of controls were normalized to 1.

### Western analysis

The melanoma tumor tissues and cultured cells were harvested in Tween 20 buffer containing 50mM HEPES (pH 8.0), 150mM NaCl, 2.5mM EGTA, 1mM EDTA, 0.1% Tween 20, and protease/phosphatase inhibitors (1mM phenylmethylsulphonyl fluoride, 20 U of aprotinin/ml, 5mg of leupeptin/ml, 1mM DTT, 0.4mM NaF, and 10mM β-glycerophosphate). Lysates were sonicated prior to clearing by centrifugation at 4°C for 30 min. Proteins were resolved by SDS-PAGE, transferred to membrane, and subjected to immunoblot. Antibodies utilized include PERK (Rockland), p-eIF2α S51 (Cell Signaling), BiP (Cell Signaling), total eIF2α (Cell Signaling), Cyclin D1 (mouse monoclonal D1-72-13G), Cul4a (Bethyl, A300-739A), p-AktS473 (Cell Signaling), total Akt (Cell Signaling), GAPDH (Cell Signaling) and β-actin (Sigma Aldrich).

### Senescence-associated β-galactosidase

Tumor tissue samples were harvested from mice and snap frozen in tissue-Tek O.C.T. 6-micron thick frozen tissue sections were prepared according to standard procedures (Sigma, Senescence-Galactosidase Staining Kit #9860).

### Histology

10% buffered formalin was used to fix tissues (overnight), followed by dehydration with ethanol, paraffin embedding, and sectioning. 5- to 8-μm sections were used for immunohistochemistry (IHC), sections were dewaxed and rehydrated in gradient ethanol followed by melanin depigmentation. Sections were immersed in 10% hydrogen peroxide and boiled for 20 min at 65°C. After microscopic inspection, the sections were rinsed with tap water for 5 min. Standard protocols were utilized for hematoxylin and eosin (H&E) staining. Antibodies utilized for IHC include: cyclin D1 (mouse monoclonal D1-72-13G), S-100 (Dako), CHOP (Cell Signaling), p-eIF2α (Cell Signaling), pAkt (Cell Signaling), pS6 (Cell Signaling), γH2Ax (Cell Signaling), pATM (Cell Signaling), p21 (Santa Cruz Biotechnology), H4R3me2 (Epigenetic), pRb (Santa Cruz Biotechnology), CD31 (Cell Signaling), pERK (Cell Signaling), pH3 (Cell Signaling), Carbonic anhydrase IX/CA9 (Novus Biologicals). Antigens were retrieved with Antigen Retrieval Citra Plus (Biogenic) by boiling for 15 min, and antibodies were visualized with a Vectastain ABC Elite kit (Vector Laboratories) and a peroxidase substrate kit DAB (Vector Laboratories). Sections were also tested for apoptosis by using TdT In Situ Apoptosis Detection Kit—DAB (R&D Systems). For quantification of IHC, the average percentage of positively stained cell evaluated form each section were scored according to staining index (SI) scale (- = no stain; + = < 33%; ++ = 33%-66%; +++ = >66%). Representative fields from each sections were chosen are presented in figures.

### Soft agar assays

Soft agar assays, were performed in 6-well plates (2500 cells seeded) containing 0.4% low melting point agarose (Lonza) lower layer, and on top of 0.8% agarose-top layer. Cells were growth in 37°C, 5%CO_2_ for 21–26 days and colonies were quantified.

### Statistics

GraphPad Prism software was utilized to analyze Kaplan-Meier tumor-free survival graphs. A two-tailed Student t test was utilized for other statistical analyses (P values of <0.05 indicating statistical significance). Error bars in the figures represent.

## Supporting Information

S1 Text Supplemental Methods. (PDF)

S1 Fig Quantification of beta-galactosidase assay in premalignant skin isolated from Braf^V600E^, Perk +/- and -/- mice related to Fig 3. (PDF)

S2 Fig Related to Fig 4. A-B)TREESpot visualization of kinase specificity of LY-4 200nM (A), 2000nM (B) against 456 kinases. Images were generated using the TREEspot software tool and reprinted with permission from KINOMEscan, a division of DiscoveRx Corporation, DISCOVERX CORPORATION 2010.
(PDF)

S3 Fig Related to Fig 4. **A)** H&E and IHC analysis of Braf^V600ECA/+^/Perk+/- skin +/-LY-4, scale bars, 50 mm. **B)** H&E of pancreas from control or LY-4 treated mice. **C)** LY-4 does not inhibit MAPK. **D)** Blood glucose levels +/- LY4; p-values analyzed by two-tailed Student t test. **E)** Survival curve of TyrCre; BrafCA/+ mice post LY-4 tretement.
(PDF)

S4 Fig Analysis of premalignant skin from Braf^V600ECA/+^,PTEN-/- mice treated with PERK specific inhibitor LY-4 and blood glucose analyze, Related to Fig 5. A) Braf^V600ECA/+^/Perk+/- mice develop melanoma. B) H&E and IHC analysis of Braf^V600E^, PTEN-/- mice skin +/- LY-4, scale bars, 50 mm. C) Blood glucose measurement and the end of the experiment (Control mice n = 5, LY-4 treated mice n = 4; p-values analyzed by two-tailed Student t test). D) Pancreas following 25 days of LY4 treatment. E) Weight of tumors +/- LY4; p-values analyzed by two-tailed Student t test.
(PDF)

S5 Fig PERK inhibition decreases survival of human melanoma cell lines exposed to ER stress. Related to Fig 6. A) Western blot of melanoma cell lysates treated with PERK inhibitor GSK2656157 +/- thapsigargin (Tg). B) Clonogenic survival of human melanoma cell lines treated with thapsigargin +/- PERK inhibitors LY-4 and GSK2656157.
(PDF)

S6 Fig Perk mutants posses ability to form colonies and localize into ER, Related to Fig 7. **A)** Focus assay of Perk mutants in MEF’s stabile cell lines (Giemsa staining) **B)** Fluorescence localization assay Perk (green), ER (red), scale bars, 20 mm.
(PDF)

S1 Table LY-4 and GSK2656157 selectivity towards other eIF2α kinases. (PDF)

S2 Table DiscoverX Kinase Selectivity analyze. (PDF)

S3 Table p53 mutations detected in the Perk+/- mouse melanomas. (PDF)
