# Supplementary material for: PERK Is a Haploinsufficient Tumor Suppressor: Gene Dose Determines Tumor-Suppressive Versus Tumor Promoting Properties of PERK in Melanoma
Source: PLoS Genet. 2016 Dec 15;12(12):e1006518. doi: 10.1371/journal.pgen.1006518 (PMC5207760; doi:10.1371/journal.pgen.1006518)
Supplement: S1 Text — (PDF) [file pgen.1006518.s001.pdf]

S1. Text. Supplemental Methods

Biochemical Assays related to assessment of LY-4 selectivity for PERK. Recombinant human EIF2AK3 (PERK) catalytic domain (amino acids 536 – 1116), GFP-eIF2α substrate, and Terbium-labelled phospho-eIF2α antibody were purchased from Invitrogen (Carlsbad, CA). HIS-SUMO-GCN2 catalytic domain (amino acids 584 – 1019) was expressed and purified from *E. coli*. TR-FRET kinase assays were performed in the absence or presence of inhibitors in a reaction buffer consisting of 50 mM HEPES, pH 7.5, 10 mM $MgCl_2$, 1.0 mM EGTA, and 0.01% Brij-35, and 100 – 200 nM GFP-eIF2α substrate. PERK assays contained 62.5 ng/ml enzyme and 1.5 µM ATP ($Km_{, app}$ ~1.5 uM) and GCN2 assays contained 3 nM enzyme and 90 µM ATP ($Km_{, app}$ ~200 uM). Following addition of test compound, the reaction was initiated by addition of enzyme and incubated at room temperature for 45 minutes. The reaction was stopped by addition of EDTA to a final concentration of 10 mM and Terbium-labeled phospho-eIF2α antibody was added at a final concentration of 2 nM and incubated for 90 minutes. The resulting fluorescence was monitored in an EnVison® Multilabel reader (PerkinElmer, Waltham, MA). TR-FRET ratios and the resulting IC_{50} values were determined from the fitted inhibition curves. Biochemical specificity profiling was performed at DiscoveRx (San Diego, CA) according to the manufacturer's protocol.

Cell-based TR-FRET assay for IC50 evaluation. A detailed description of the development of this assay will be published elsewhere. Briefly, GripTite™ 293 cells (Invitrogen, Carlsbad, CA) expressing GFP-eIF2α were seeded at 10,000 cells per well in 384-well plates and allowed to attach overnight. Cells were pre-treated with test compounds for 1 hour. Tunicamycin (1 µM) was added to induce PERK activity and the plates were incubated at 37 °C for 2 hours. The culture media was removed and the cells were lysed in buffer consisting of 20 mM Tris-HCl, pH 7.5, 150 mM NaCl, 5 mM EDTA, 1% NP-40, 5 mM NaF, Protease inhibitors (Sigma, St. Louis, MO), Phosphatase inhibitors (Sigma, St. Louis, MO), and 2 nM Terbium-labelled anti-phospho-eIF2 antibody (Invitrogen, Carlsbad, CA). Cell lysates were incubated for 2 hours in the dark at room temperature and fluorescence was monitored in an EnVison® Multilabel reader (PerkinElmer, Waltham, MA). TR-FRET ratios and the resulting IC_{50} values were determined from the fitted inhibition curves using un-induced (100% inhibition) and induced (0% inhibition) wells as controls.

Plasmids, Lentiviral Production and Infection

The pLU-EF1a-MCS-PGK-Blasticidin and pLU-EF1a-BRAFV600E-PGK-Blasticidin plasmids were generated in the Protein Expression Core at the Wistar Institute. 293T cells were co-transfected with packaging and envelope plasmids, pCMV and pVsvg with pLU-EF1a-MCS-PGK-Blasticidin or pLU-EF1a-BRAFV600E-PGK-Blasticidin and Lipofectamine 2000 (Life Technologies). Supernatants containing lentiviral particles were collected every 24 h for 3 days, and were then pooled, filtered and aliquoted. Vials of lentiviral particles were stored in a -80°C freezer. Adherent skin primary melanocytes were infected overnight with lentiviral particles supplemented with polybrene (Sigma) at 8 µg/ml. Stably infected clones were established by an initial selection with blasticidin at 8 µg/ml for 6 days.

Cell culture and plasmids

Human primary melanocytes isolated from foreskin specimen. The cells were maintained in 254CF media (M-254CF, Gibco) containing HMGS-2 (S-016-5, Gibco) and infected with the lentivirus generated from the plasmids plu-EF1a-$Braf^{V600E}$ and control plu-EF1a-MCS. Mouse embryonic fibroblasts (MEFs) were grown in Dulbecco's modified Eagle's medium (DMEM) (high-glucose formulation) with 4mM L-glutamine, 10% (v/v) fetal bovine serum, 100U/mL penicillin, 100µg/mL streptomycin, and 55mM β-mercaptoethanol.

Supplemental Figure Legends

S1 Fig. Quantification of b-galactosidase assay in premalignant skin isolated from Braf^{V600E}, Perk +/- and -/- mice related to Figure 3.

S2 Fig. Skin analyzes of Braf$^{V600ECA/+}$, PTEN-/- mice treated with PERK specific inhibitor LY-4 and blood glucose analyze, Related to Figure 4.
A-B)TREESpot™ visualization of kinase specificity of LY-4 200nM (G), 2000nM (H) against 456 kinases. Images were generated using the TREEspot™ software tool and reprinted with permission from KINOMEscan®, a division of DiscoveRx Corporation, © DISCOVERX CORPORATION 2010.

S3 Fig. Related to Figure 4.
A) H&E and IHC analysis of Braf$^{V600ECA/+}$/Perk+/- skin +/-LY-4, scale bars, 50 mm. **B)** H&E of pancreas from control or LY-4 treated mice. **C)** LY-4 does not inhibit MAPK. **D)** Blood glucose levels +/- LY4; ; p-values analyzed by two-tailed Student t test. **E)** Survival curve of TyrCre; BrafCA/+ mice post LY-4 tretement.

S4 Fig. Analysis of premalignant skin from Braf$^{V600ECA/+}$,PTEN-/- mice treated with PERK specific inhibitor LY-4 and blood glucose analyze, Related to Figure 5.
A) Braf$^{V600ECA/+}$/Perk+/- mice develop melanoma. B) H&E and IHC analysis of Braf^{V600E}, PTEN-/- mice skin +/- LY-4, scale bars, 50 mm. C) Blood glucose measurement and the end of the experiment (Control mice n=5, LY-4 treated mice n=4; p-values analyzed by two-tailed Student t test). D) Pancreas following 25 days of LY4 treatment. E) Weight of tumors +/- LY4 ; p-values analyzed by two-tailed Student t test

S5 Fig. PERK inhibition decreases survival of human melanoma cell lines exposed to ER stress. Related to Figure 6.
A) Western blot of melanoma cell lysates treated with PERK inhibitor GSK2656157 +/- thapsigargin (Tg)
B) Clonogenic survival of human melanoma cell lines treated with thapsigargin +/- PERK inhibitors LY-4 and GSK2656157.

S6 Fig. Perk mutants posses ability to form colonies and localize into ER, Related to Figure 7.
A) Focus assay of Perk mutants in MEF's stabile cell lines (Giemsa staining) **B)** Fluorescence localization assay Perk (green), ER (red), scale bars, 20 mm.

Supplemental Table Legends

S1 Table. LY-4 and GSK2656157 selectivity towards other eIF2a kinases
S2 Table. DiscoverX Kinase Selectivity analyze
S3 Table. p53 mutations detected in the Perk+/- mouse melanomas
