# Supplementary material for: PERK Is a Haploinsufficient Tumor Suppressor: Gene Dose Determines Tumor-Suppressive Versus Tumor Promoting Properties of PERK in Melanoma
Source: PLoS Genet. 2016 Dec 15;12(12):e1006518. doi: 10.1371/journal.pgen.1006518 (PMC5207760; doi:10.1371/journal.pgen.1006518)
Supplement: S1 Fig — (PDF) [file pgen.1006518.s002.pdf]

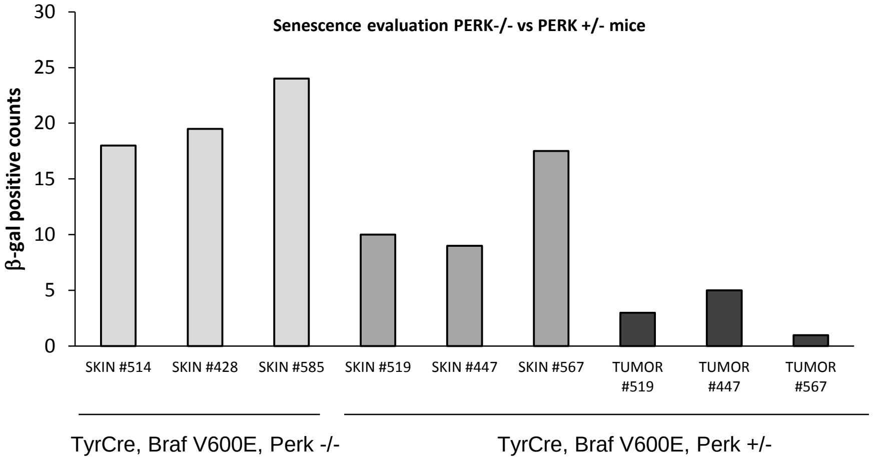

S1 Fig. Quantification of β-galactosidase assay in premalignant skin isolated from Braf^{V600E}, Perk +/- and -/- mice related to Figure 3.
