# Supplementary material for: PERK Is a Haploinsufficient Tumor Suppressor: Gene Dose Determines Tumor-Suppressive Versus Tumor Promoting Properties of PERK in Melanoma
Source: PLoS Genet. 2016 Dec 15;12(12):e1006518. doi: 10.1371/journal.pgen.1006518 (PMC5207760; doi:10.1371/journal.pgen.1006518)
Supplement: S2 Fig — A-B)TREESpot visualization of kinase specificity of LY-4 200nM (A), 2000nM (B) against 456 kinases. Images were generated using the TREEspot software tool and reprinted with permission from KINOMEscan, a division of DiscoveRx Corporation, DISCOVERX CORPORATION 2010. (PDF) [file pgen.1006518.s003.pdf]

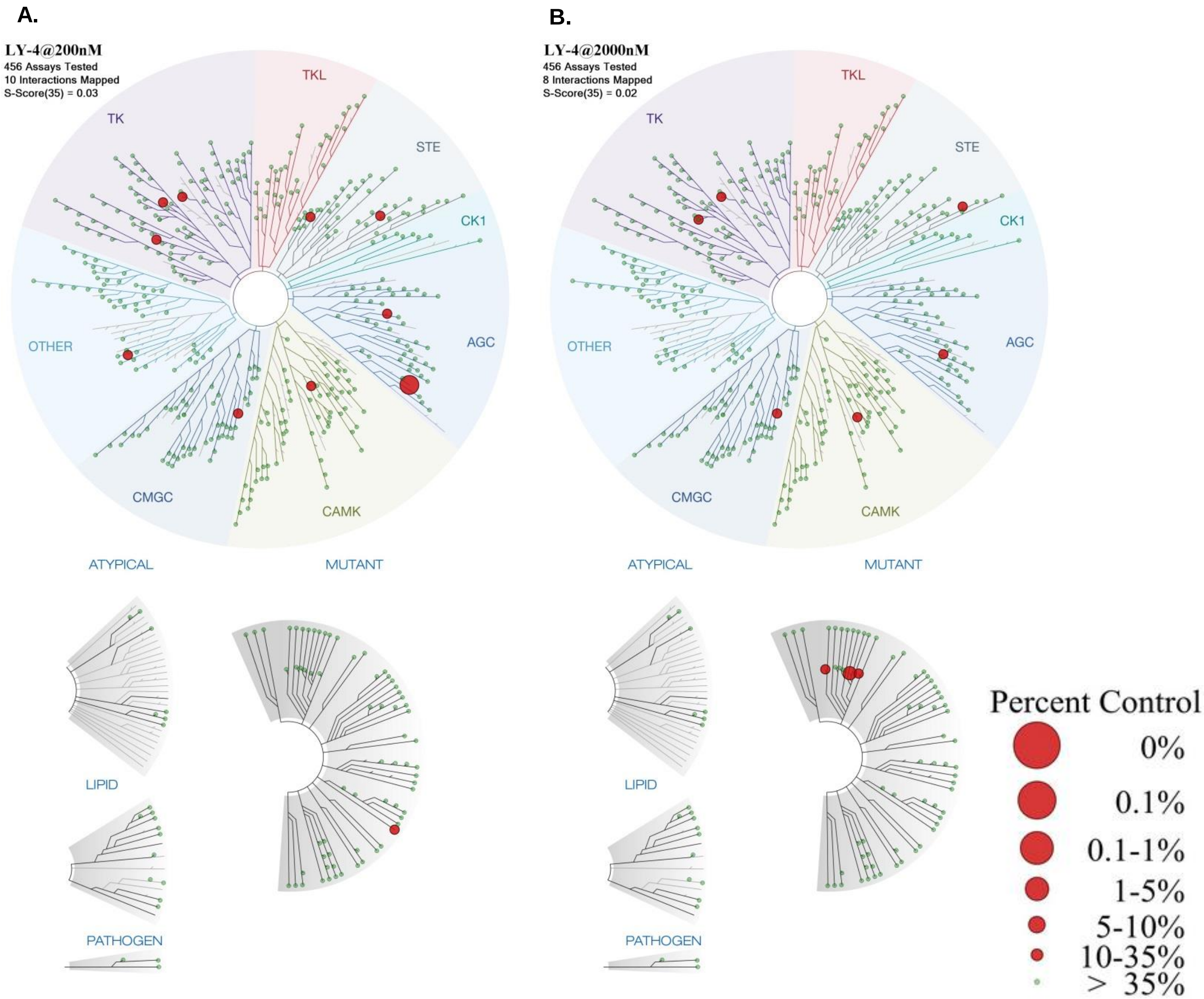

S2 Fig. Related to Figure 4. A-B)TREESpot™ visualization of kinase specificity of LY-4 200nM (A), 2000nM (B) against 456 kinases. Images were generated using the TREEspot™ software tool and reprinted with permission from KINOMEscan®, a division of DiscoveRx Corporation, © DISCOVERX CORPORATION 2010.
