# Supplementary material for: PERK Is a Haploinsufficient Tumor Suppressor: Gene Dose Determines Tumor-Suppressive Versus Tumor Promoting Properties of PERK in Melanoma
Source: PLoS Genet. 2016 Dec 15;12(12):e1006518. doi: 10.1371/journal.pgen.1006518 (PMC5207760; doi:10.1371/journal.pgen.1006518)
Supplement: S3 Fig — A) H&E and IHC analysis of BrafV600ECA/+/Perk+/- skin +/-LY-4, scale bars, 50 mm. B) H&E of pancreas from control or LY-4 treated mice. C) LY-4 does not inhibit MAPK. D) Blood glucose levels +/- LY4; p-values analyzed by two-tailed Student t test. E) Survival curve of TyrCre; BrafCA/+ mice post LY-4 tretement. (PDF) [file pgen.1006518.s004.pdf]

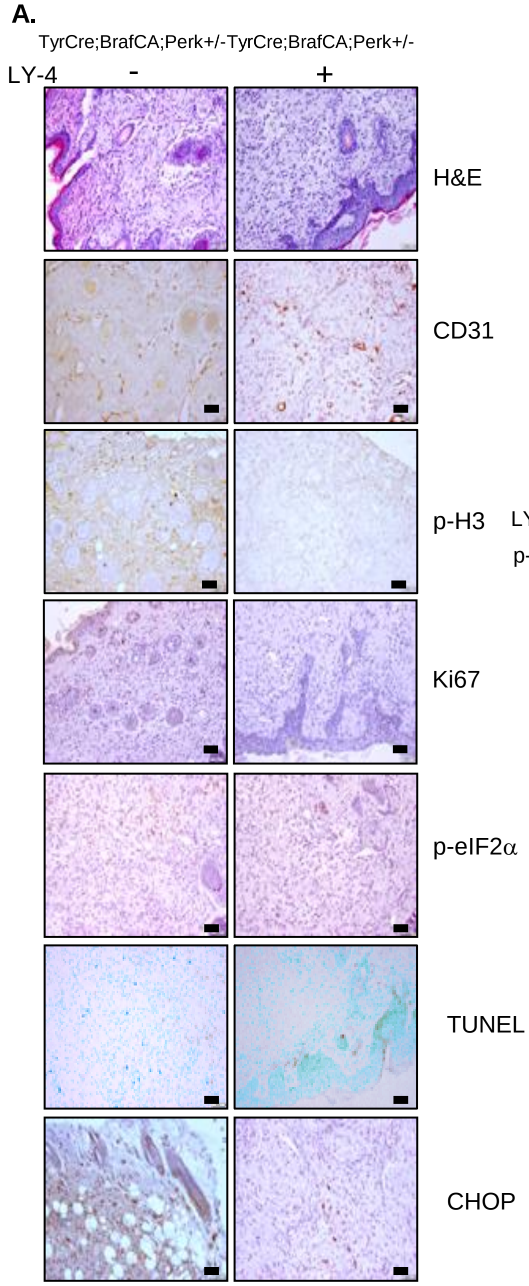

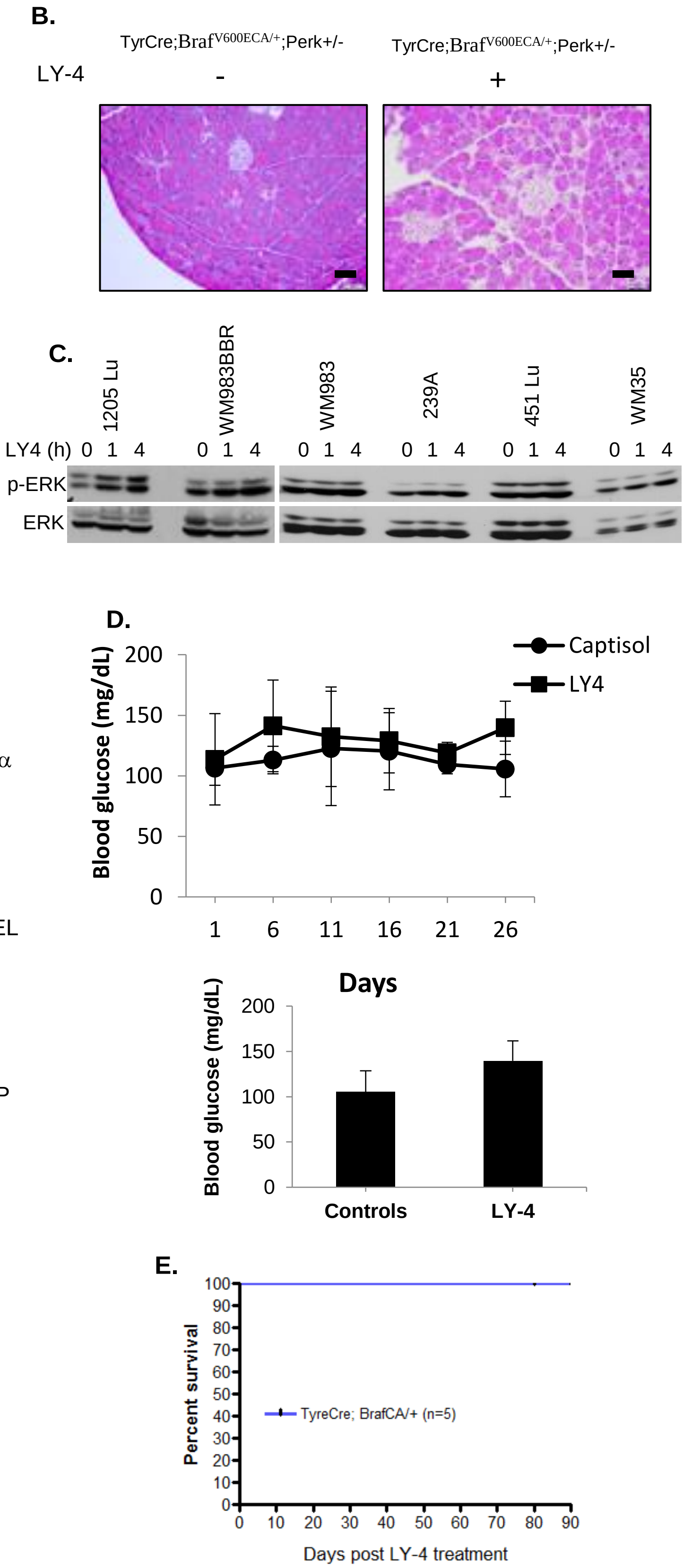

SI SCORE	Genotype	
Antibody	TyrCre;BrafCA; Perk+/-	TyrCre;BrafCA; Perk+/- + LY-4
CD31	+++	+
p-H3	++	+
Ki67	++	+
p-eIF2α	++	+
TUNEL	+	+++
CHOP	+++	+

S3 Fig. Related to Figure 4.
A) H&E and IHC analysis of Braf$^{V600ECA/+}$/Perk+/- skin +/-LY-4, scale bars, 50 μm. **B)** H&E of pancreas from control or LY-4 treated mice. **C)** LY-4 does not inhibit MAPK. **D)** Blood glucose levels +/- LY4; ; p-values analyzed by two-tailed Student t test. **E)** Survival curve of TyrCre; BrafCA/+ mice post LY-4 treatment.
