# Supplementary material for: PERK Is a Haploinsufficient Tumor Suppressor: Gene Dose Determines Tumor-Suppressive Versus Tumor Promoting Properties of PERK in Melanoma
Source: PLoS Genet. 2016 Dec 15;12(12):e1006518. doi: 10.1371/journal.pgen.1006518 (PMC5207760; doi:10.1371/journal.pgen.1006518)
Supplement: S4 Fig — A) BrafV600ECA/+/Perk+/- mice develop melanoma. B) H&E and IHC analysis of BrafV600E, PTEN-/- mice skin +/- LY-4, scale bars, 50 mm. C) Blood glucose measurement and the end of the experiment (Control mice n = 5, LY-4 treated mice n = 4; p-values analyzed by two-tailed Student t test). D) Pancreas following 25 days of LY4 treatment. E) Weight of tumors +/- LY4; p-values analyzed by two-tailed Student t test. (PDF) [file pgen.1006518.s005.pdf]

A.

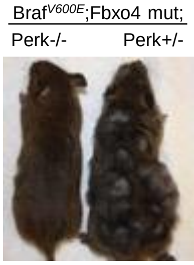

B.

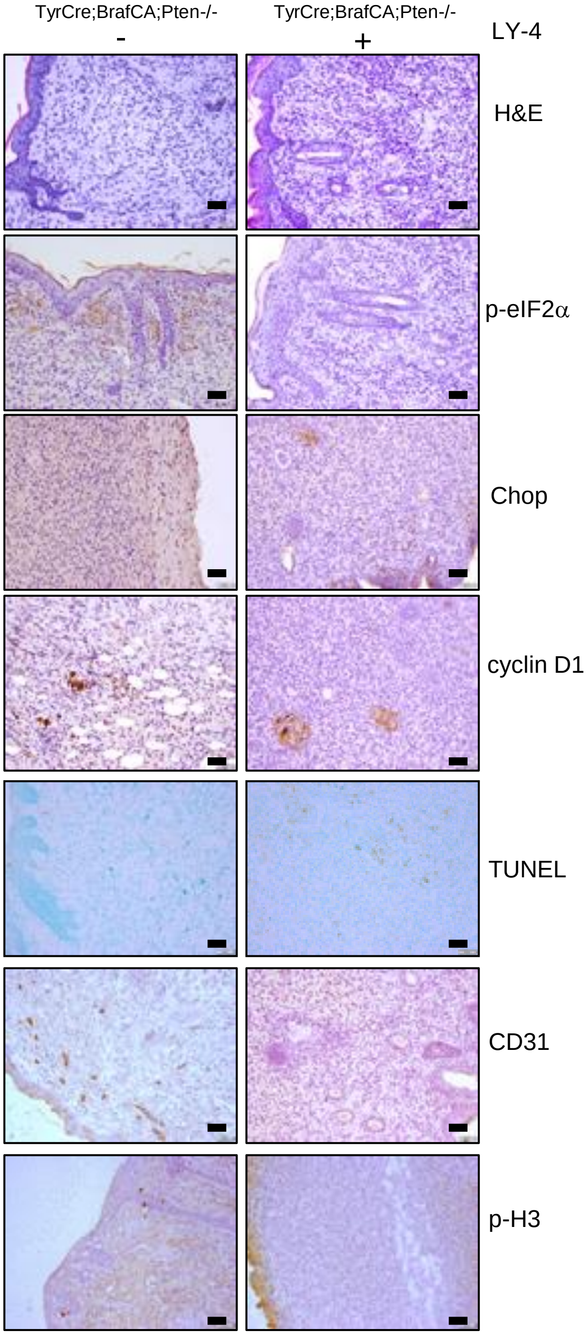

C.

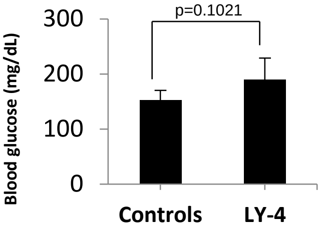

D.

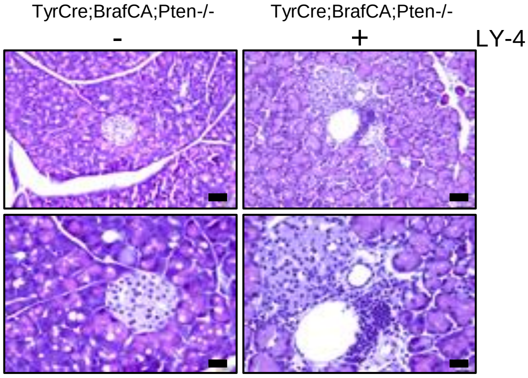

E.

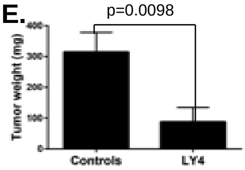

SI SCORE	Genotype	
Antibody	TyrCre;BrafCA; Pten-/-	TyrCre;BrafCA; Pten-/- + LY-4
p-eIF2α	+++	+
Chop	+++	+
cyclin D1	++	+
TUNEL	++	+++
CD31	++	+
p-H3	++	+

S4 Fig. Analysis of premalignant skin from Braf$^{V600ECA/+}$,Pten-/- mice treated with PERK specific inhibitor LY-4 and blood glucose analyze, Related to Figure 5.

A) Braf$^{V600ECA/+}$/Perk+/- mice develop melanoma. B) H&E and IHC analysis of Braf^{V600E}, PTEN-/- mice skin +/- LY-4, scale bars, 50 μm. C) Blood glucose measurement and the end of the experiment (Control mice n=5, LY-4 treated mice n=4; p-values analyzed by two-tailed Student t test). D) Pancreas following 25 days of LY4 treatment. E) Weight of tumors +/- LY4 ; p-values analyzed by two-tailed Student t test.
