# Supplementary material for: PERK Is a Haploinsufficient Tumor Suppressor: Gene Dose Determines Tumor-Suppressive Versus Tumor Promoting Properties of PERK in Melanoma
Source: PLoS Genet. 2016 Dec 15;12(12):e1006518. doi: 10.1371/journal.pgen.1006518 (PMC5207760; doi:10.1371/journal.pgen.1006518)
Supplement: S5 Fig — A) Western blot of melanoma cell lysates treated with PERK inhibitor GSK2656157 +/- thapsigargin (Tg). B) Clonogenic survival of human melanoma cell lines treated with thapsigargin +/- PERK inhibitors LY-4 and GSK2656157. (PDF) [file pgen.1006518.s006.pdf]

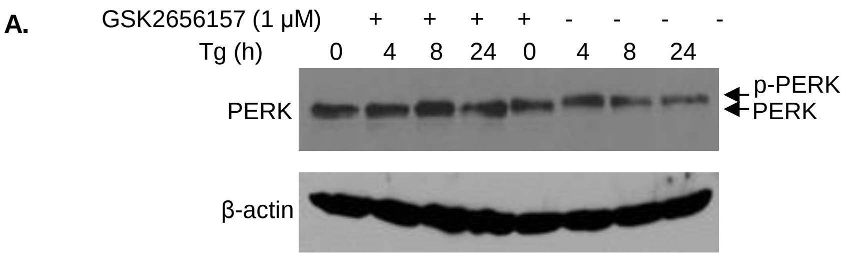

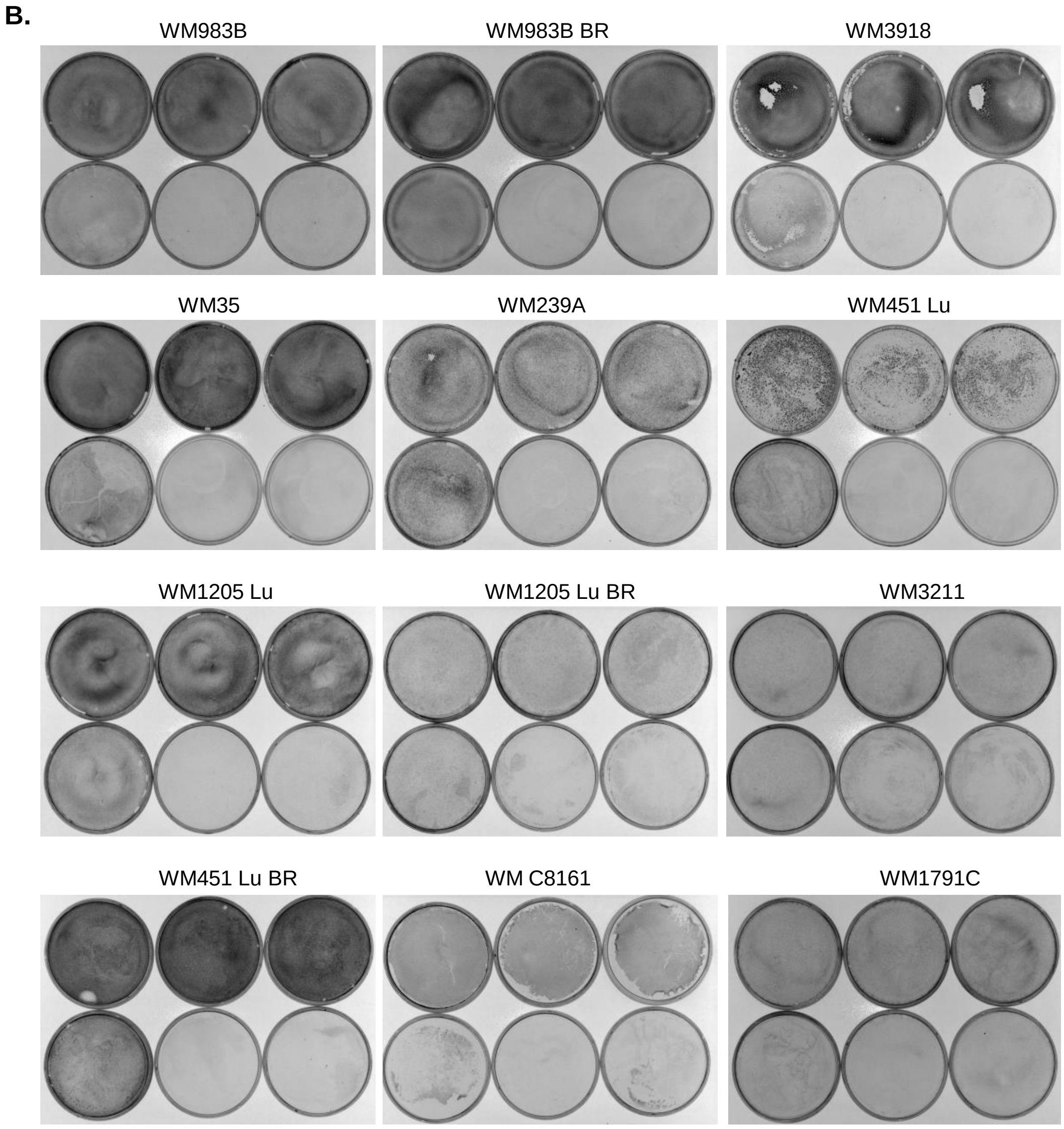

DMSO	LY-4	GSK2656157
Tg	LY-4 + Tg	GSK2656157 + Tg

S5 Fig. PERK inhibition decreases survival of human melanoma cell lines exposed to ER stress. Related to Figure 6.

A) Western blot of melanoma cell lysates treated with PERK inhibitor GSK2656157 +/- thapsigargin (Tg)

B) Clonogenic survival of human melanoma cell lines treated with thapsigargin +/- PERK inhibitors LY-4 and GSK2656157.
