# Supplementary material for: PERK Is a Haploinsufficient Tumor Suppressor: Gene Dose Determines Tumor-Suppressive Versus Tumor Promoting Properties of PERK in Melanoma
Source: PLoS Genet. 2016 Dec 15;12(12):e1006518. doi: 10.1371/journal.pgen.1006518 (PMC5207760; doi:10.1371/journal.pgen.1006518)
Supplement: S6 Fig — A) Focus assay of Perk mutants in MEF’s stabile cell lines (Giemsa staining) B) Fluorescence localization assay Perk (green), ER (red), scale bars, 20 mm. (PDF) [file pgen.1006518.s007.pdf]

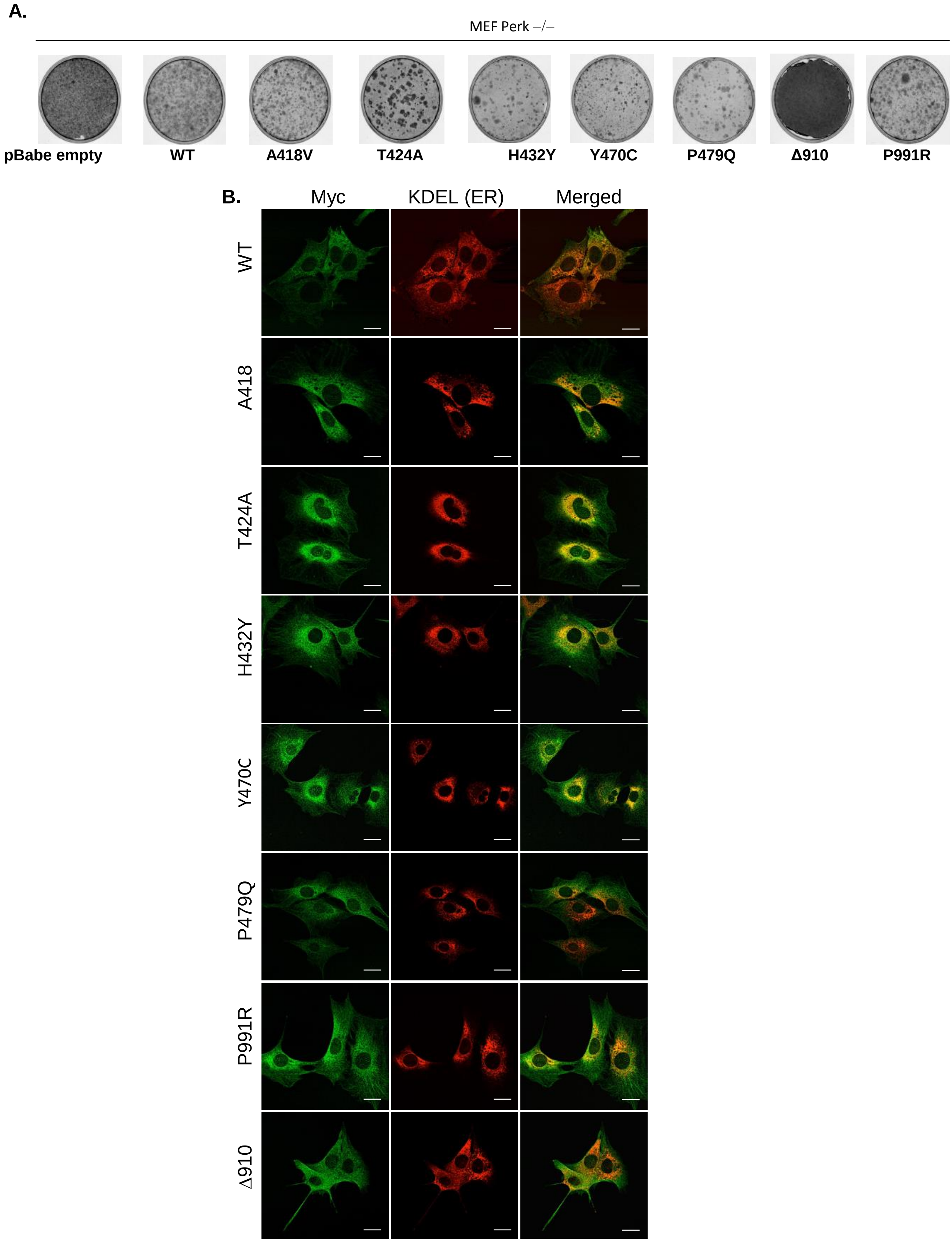

S6 Fig. Perk mutants posses ability to form colonies and localize into ER, Related to Figure 7.
A) Focus assay of Perk mutants in MEF's stabile cell lines (Giemsa staining) **B)** Fluorescence localization assay Perk (green), ER (red), scale bars, 20 μm.
