# Supplementary material for: PERK Is a Haploinsufficient Tumor Suppressor: Gene Dose Determines Tumor-Suppressive Versus Tumor Promoting Properties of PERK in Melanoma
Source: PLoS Genet. 2016 Dec 15;12(12):e1006518. doi: 10.1371/journal.pgen.1006518 (PMC5207760; doi:10.1371/journal.pgen.1006518)
Supplement: S1 Table — (PDF) [file pgen.1006518.s008.pdf]

S1 Table. LY-4 and GSK2656157 selectivity towards other eIF2α kinases

Compound	PERK[a] Enzyme IC_{50} (μM)	GCN2[a] Enzyme IC_{50} (μM)	PERK[b] Cell-based IC_{50} (μM)	ATF4-luc[c] Cell-based IC_{50} (μM)	DiscoverX Kinase Selectivity[d] (# kinases inhibited >50%)		
					0.2 μM	2.0 μM	20.0 μM
LY-4	0.002	18.1	0.054	0.048	19	20	48
GSK2656157	0.008	>200	0.036	0.021	34	80	183

[a]Activity in PERK and GCN2 biochemical kinase assays using purified eIF2α as substrate. [b]Cell-based activity in assay of tunicamycin-induced eIF2α phosphorylation in 293 cells. [c]Activity in assay of tunicamycin-induced ATF4 activity in 293 cells. [d]Summary of kinase specificity against 456 kinases at DiscoveRx.
