# Supplementary material for: PERK Is a Haploinsufficient Tumor Suppressor: Gene Dose Determines Tumor-Suppressive Versus Tumor Promoting Properties of PERK in Melanoma
Source: PLoS Genet. 2016 Dec 15;12(12):e1006518. doi: 10.1371/journal.pgen.1006518 (PMC5207760; doi:10.1371/journal.pgen.1006518)
Supplement: S2 Table — (PDF) [file pgen.1006518.s009.pdf]

S2 Table. DiscoverX Kinase Selectivity analyze

KINOMEscan Gene Symbol	Entrez Gene Symbol	LY-4		
		0.2 μM	2 μM	20 μM
AAK1	AAK1	41	25	5
ABL1(E255K)-phosphorylated	ABL1	0	51	0
ABL1(F317I)-nonphosphorylated	ABL1	33	26	22
ABL1(F317I)-phosphorylated	ABL1	30	20	18
ABL1(F317L)-nonphosphorylated	ABL1	19	23	40
ABL1(F317L)-phosphorylated	ABL1	0	0	42
ABL1(H396P)-nonphosphorylated	ABL1	0	76	29
ABL1(H396P)-phosphorylated	ABL1	0	41	0
ABL1(M351T)-phosphorylated	ABL1	0	45	25
ABL1(Q252H)-nonphosphorylated	ABL1	46	91	0
ABL1(Q252H)-phosphorylated	ABL1	39	43	32
ABL1(T315I)-nonphosphorylated	ABL1	30	7	5
ABL1(T315I)-phosphorylated	ABL1	0	0	0
ABL1(Y253F)-phosphorylated	ABL1	0	26	29
ABL1-nonphosphorylated	ABL1	10	69	3
ABL1-phosphorylated	ABL1	0	30	32
ABL2	ABL2	0	0	0
ACVR1	ACVR1	0	4	57
ACVR1B	ACVR1B	27	14	0
ACVR2A	ACVR2A	3	45	68
ACVR2B	ACVR2B	0	45	68
ACVRL1	ACVRL1	0	18	12
ADCK3	CABC1	0	7	29
ADCK4	ADCK4	28	22	23
AKT1	AKT1	0	5	0
AKT2	AKT2	0	0	0
AKT3	AKT3	0	0	13
ALK	ALK	0	0	77
ALK(C1156Y)	ALK	0	0	15
ALK(L1196M)	ALK	28	0	36
AMPK-alpha1	PRKAA1	0	0	0
AMPK-alpha2	PRKAA2	0	0	7
ANKK1	ANKK1	29	14	48
ARK5	NUAK1	0	0	23
ASK1	MAP3K5	39	45	31
ASK2	MAP3K6	0	0	0
AURKA	AURKA	0	32	47
AURKB	AURKB	0	0	2
AURKC	AURKC	18	3	21
AXL	AXL	20	7	0
BIKE	BMP2K	14	0	30
BLK	BLK	20	12	45
BMPR1A	BMPR1A	53	50	61
BMPR1B	BMPR1B	0	26	81
BMPR2	BMPR2	9	7	26
BMX	BMX	8	31	34
BRAF	BRAF	0	13	19
BRAF(V600E)	BRAF	0	12	30
BRK	PTK6	0	0	3
BRSK1	BRSK1	0	1	5
BRSK2	BRSK2	9	23	0
BTK	BTK	0	0	0
BUB1	BUB1	18	0	0
CAMK1	CAMK1	39	16	43
CAMK1D	CAMK1D	20	12	25
CAMK1G	CAMK1G	34	23	0
CAMK2A	CAMK2A	13	0	64

CAMK2B	CAMK2B	37	0	70
CAMK2D	CAMK2D	18	0	5
CAMK2G	CAMK2G	40	43	28
CAMK4	CAMK4	0	18	0
CAMKK1	CAMKK1	0	0	0
CAMKK2	CAMKK2	56	52	24
CASK	CASK	73	9	42
CDC2L1	CDK11B	0	0	14
CDC2L2	CDC2L2	27	10	24
CDC2L5	CDK13	0	4	7
CDK11	CDK19	1	2	0
CDK2	CDK2	16	12	0
CDK3	CDK3	6	14	25
CDK4-cyclinD1	CDK4	0	4	5
CDK4-cyclinD3	CDK4	0	41	24
CDK5	CDK5	0	0	0
CDK7	CDK7	0	0	0
CDK8	CDK8	30	0	0
CDK9	CDK9	0	0	0
CDKL1	CDKL1	31	0	0
CDKL2	CDKL2	2	0	0
CDKL3	CDKL3	9	0	0
CDKL5	CDKL5	17	0	0
CHEK1	CHEK1	18	44	21
CHEK2	CHEK2	0	0	1
CIT	CIT	5	0	0
CLK1	CLK1	7	8	45
CLK2	CLK2	6	0	35
CLK3	CLK3	15	15	0
CLK4	CLK4	4	0	34
CSF1R	CSF1R	0	5	0
CSF1R-autoinhibited	CSF1R	0	0	0
CSK	CSK	31	0	14
CSNK1A1	CSNK1A1	2	0	53
CSNK1A1L	CSNK1A1L	0	15	64
CSNK1D	CSNK1D	27	17	26
CSNK1E	CSNK1E	0	0	17
CSNK1G1	CSNK1G1	2	0	8
CSNK1G2	CSNK1G2	12	39	63
CSNK1G3	CSNK1G3	17	0	14
CSNK2A1	CSNK2A1	66	58	70
CSNK2A2	CSNK2A2	0	26	46
CTK	MATK	35	0	18
DAPK1	DAPK1	0	15	12
DAPK2	DAPK2	22	32	56
DAPK3	DAPK3	14	0	0
DCAMKL1	DCLK1	0	78	47
DCAMKL2	DCLK2	0	0	0
DCAMKL3	DCLK3	0	0	26
DDR1	DDR1	18	27	17
DDR2	DDR2	4	0	0
DLK	MAP3K12	0	0	0
DMPK	DMPK	14	19	56
DMPK2	CDC42BPG	0	0	14
DRAK1	STK17A	0	0	0
DRAK2	STK17B	22	0	0
DYRK1A	DYRK1A	26	11	45
DYRK1B	DYRK1B	25	19	35
DYRK2	DYRK2	18	0	20
EGFR	EGFR	30	23	16
EGFR(E746-A750del)	EGFR	33	38	55
EGFR(G719C)	EGFR	0	0	0

EGFR(G719S)	EGFR	0	0	0
EGFR(L747-E749del, A750P)	EGFR	48	25	32
EGFR(L747-S752del, P753S)	EGFR	39	30	43
EGFR(L747-T751del,Sins)	EGFR	0	3	16
EGFR(L858R)	EGFR	18	25	30
EGFR(L858R,T790M)	EGFR	16	30	28
EGFR(L861Q)	EGFR	0	0	5
EGFR(S752-I759del)	EGFR	0	0	0
EGFR(T790M)	EGFR	0	0	0
EIF2AK1	EIF2AK1	0	0	35
EPHA1	EPHA1	37	36	53
EPHA2	EPHA2	18	21	40
EPHA3	EPHA3	13	16	0
EPHA4	EPHA4	34	0	31
EPHA5	EPHA5	0	0	0
EPHA6	EPHA6	15	0	43
EPHA7	EPHA7	1	0	6
EPHA8	EPHA8	6	0	0
EPHB1	EPHB1	4	2	1
EPHB2	EPHB2	4	0	12
EPHB3	EPHB3	7	1	16
EPHB4	EPHB4	24	0	4
EPHB6	EPHB6	20	7	42
ERBB2	ERBB2	0	0	0
ERBB3	ERBB3	0	0	0
ERBB4	ERBB4	0	0	4
ERK1	MAPK3	2	18	0
ERK2	MAPK1	0	0	1
ERK3	MAPK6	0	32	3
ERK4	MAPK4	0	0	0
ERK5	MAPK7	0	0	4
ERK8	MAPK15	0	0	0
ERN1	ERN1	0	9	40
FAK	PTK2	8	10	0
FER	FER	11	18	10
FES	FES	13	0	23
FGFR1	FGFR1	5	0	0
FGFR2	FGFR2	0	15	19
FGFR3	FGFR3	27	29	30
FGFR3(G697C)	FGFR3	32	8	24
FGFR4	FGFR4	24	6	3
FGR	FGR	11	0	22
FLT1	FLT1	27	18	35
FLT3	FLT3	0	0	23
FLT3(D835H)	FLT3	57	2	20
FLT3(D835Y)	FLT3	17	3	22
FLT3(ITD)	FLT3	0	0	18
FLT3(K663Q)	FLT3	0	0	15
FLT3(N841I)	FLT3	60	53	59
FLT3(R834Q)	FLT3	4	3	6
FLT3-autoinhibited	FLT3	36	0	0
FLT4	FLT4	30	4	0
FRK	FRK	8	6	12
FYN	FYN	8	0	12
GAK	GAK	0	0	11
GCN2(Kin.Dom.2,S808G)	EIF2AK4	0	0	28
GRK1	GRK1	70	54	78
GRK4	GRK4	13	1	0
GRK7	GRK7	0	0	0
GSK3A	GSK3A	0	0	0
GSK3B	GSK3B	0	17	41
HASPIN	GSG2	9	46	84

HCK	HCK	0	0	16
HIPK1	HIPK1	0	21	33
HIPK2	HIPK2	0	0	45
HIPK3	HIPK3	0	4	43
HIPK4	HIPK4	22	53	83
HPK1	MAP4K1	50	0	32
HUNK	HUNK	0	0	0
ICK	ICK	34	6	46
IGF1R	IGF1R	0	0	0
IKK-alpha	CHUK	0	0	15
IKK-beta	IKBKB	0	0	0
IKK-epsilon	IKBKE	43	0	19
INSR	INSR	0	0	0
INSRR	INSRR	33	14	40
IRAK1	IRAK1	0	0	6
IRAK3	IRAK3	24	40	61
IRAK4	IRAK4	0	0	2
ITK	ITK	10	8	0
JAK1(JH1domain-catalytic)	JAK1	0	0	0
JAK1(JH2domain-pseudokinase)	JAK1	78	67	11
JAK2(JH1domain-catalytic)	JAK2	26	42	36
JAK3(JH1domain-catalytic)	JAK3	24	0	0
JNK1	MAPK8	0	2	8
JNK2	MAPK9	11	0	24
JNK3	MAPK10	5	0	41
KIT	KIT	9	23	19
KIT(A829P)	KIT	0	0	0
KIT(D816H)	KIT	0	0	0
KIT(D816V)	KIT	0	16	42
KIT(L576P)	KIT	6	30	21
KIT(V559D)	KIT	0	26	36
KIT(V559D,T670I)	KIT	0	6	20
KIT(V559D,V654A)	KIT	23	0	0
KIT-autoinhibited	KIT	69	52	5
LATS1	LATS1	6	7	7
LATS2	LATS2	0	0	14
LCK	LCK	9	0	5
LIMK1	LIMK1	0	0	0
LIMK2	LIMK2	20	0	30
LKB1	STK11	22	0	31
LOK	STK10	0	0	53
LRRK2	LRRK2	2	6	8
LRRK2(G2019S)	LRRK2	0	0	0
LTK	LTK	38	0	45
LYN	LYN	19	14	26
LZK	MAP3K13	0	0	0
MAK	MAK	0	0	0
MAP3K1	MAP3K1	14	0	25
MAP3K15	MAP3K15	4	3	27
MAP3K2	MAP3K2	13	66	91
MAP3K3	MAP3K3	0	12	78
MAP3K4	MAP3K4	37	20	37
MAP4K2	MAP4K2	53	0	65
MAP4K3	MAP4K3	4	5	50
MAP4K4	MAP4K4	3	14	8
MAP4K5	MAP4K5	0	29	31
MAPKAPK2	MAPKAPK2	17	11	0
MAPKAPK5	MAPKAPK5	0	0	0
MARK1	MARK1	15	0	0
MARK2	MARK2	0	0	0
MARK3	MARK3	0	0	0
MARK4	MARK4	19	0	0

MAST1	MAST1	9	0	34
MEK1	MAP2K1	0	0	11
MEK2	MAP2K2	0	0	21
MEK3	MAP2K3	9	22	20
MEK4	MAP2K4	73	36	62
MEK5	MAP2K5	34	24	35
MEK6	MAP2K6	9	0	14
MELK	MELK	56	29	45
MERTK	MERTK	2	7	45
MET	MET	0	12	5
MET(M1250T)	MET	18	14	0
MET(Y1235D)	MET	21	25	0
MINK	MINK1	38	21	68
MKK7	MAP2K7	0	0	17
MKNK1	MKNK1	0	0	2
MKNK2	MKNK2	0	0	21
MLCK	MYLK3	6	0	0
MLK1	MAP3K9	0	0	0
MLK2	MAP3K10	0	0	0
MLK3	MAP3K11	0	0	0
MRCKA	CDC42BPA	0	0	0
MRCKB	CDC42BPB	4	6	10
MST1	STK4	0	0	18
MST1R	MST1R	0	0	0
MST2	STK3	15	33	27
MST3	STK24	35	46	47
MST4	MST4	0	0	0
MTOR	MTOR	0	0	0
MUSK	MUSK	10	20	19
MYLK	MYLK	1	2	9
MYLK2	MYLK2	4	16	0
MYLK4	MYLK4	0	0	0
MYO3A	MYO3A	0	44	48
MYO3B	MYO3B	0	0	12
NDR1	STK38	0	0	0
NDR2	STK38L	0	0	0
NEK1	NEK1	0	5	22
NEK10	NEK10	2	15	59
NEK11	NEK11	0	8	1
NEK2	NEK2	0	0	2
NEK3	NEK3	34	59	41
NEK4	NEK4	2	7	5
NEK5	NEK5	0	0	0
NEK6	NEK6	0	0	0
NEK7	NEK7	6	27	0
NEK9	NEK9	0	1	8
NIK	MAP3K14	0	0	0
NIM1	MGC42105	23	46	55
NLK	NLK	0	0	0
OSR1	OXSR1	3	13	6
p38-alpha	MAPK14	0	0	0
p38-beta	MAPK11	30	27	0
p38-delta	MAPK13	0	0	0
p38-gamma	MAPK12	0	3	5
PAK1	PAK1	8	12	24
PAK2	PAK2	27	0	2
PAK3	PAK3	22	0	2
PAK4	PAK4	30	21	11
PAK6	PAK6	39	15	19
PAK7	PAK7	10	13	0
PCTK1	CDK16	16	18	2
PCTK2	CDK17	0	0	0

PCTK3	CDK18	5	10	0
PDGFRA	PDGFRA	26	0	0
PDGFRB	PDGFRB	0	33	24
PDPK1	PDPK1	1	0	0
PFCDPK1(P.falciparum)	CDPK1	42	0	47
PFPK5(P.falciparum)	MAL13P1.279	30	0	16
PFTAIRE2	CDK15	0	17	20
PFTK1	CDK14	0	0	0
PHKG1	PHKG1	13	18	0
PHKG2	PHKG2	0	0	17
PIK3C2B	PIK3C2B	40	39	94
PIK3C2G	PIK3C2G	18	24	15
PIK3CA	PIK3CA	9	0	8
PIK3CA(C420R)	PIK3CA	20	0	29
PIK3CA(E542K)	PIK3CA	15	0	52
PIK3CA(E545A)	PIK3CA	0	0	19
PIK3CA(E545K)	PIK3CA	34	10	29
PIK3CA(H1047L)	PIK3CA	0	0	35
PIK3CA(H1047Y)	PIK3CA	36	0	62
PIK3CA(I800L)	PIK3CA	9	6	59
PIK3CA(M1043I)	PIK3CA	0	0	0
PIK3CA(Q546K)	PIK3CA	26	12	0
PIK3CB	PIK3CB	0	0	12
PIK3CD	PIK3CD	0	0	0
PIK3CG	PIK3CG	24	0	26
PIK4CB	PI4KB	15	25	36
PIM1	PIM1	0	0	0
PIM2	PIM2	0	1	0
PIM3	PIM3	0	0	0
PIP5K1A	PIP5K1A	52	35	25
PIP5K1C	PIP5K1C	0	0	0
PIP5K2B	PIP4K2B	18	19	4
PIP5K2C	PIP4K2C	0	0	0
PKAC-alpha	PRKACA	4	0	0
PKAC-beta	PRKACB	15	0	0
PKMYT1	PKMYT1	0	17	2
PKN1	PKN1	4	25	42
PKN2	PKN2	98	4	12
PKNB(M.tuberculosis)	pknB	0	0	0
PLK1	PLK1	32	35	29
PLK2	PLK2	21	26	40
PLK3	PLK3	39	49	33
PLK4	PLK4	17	56	54
PRKCD	PRKCD	13	34	11
PRKCE	PRKCE	0	0	0
PRKCH	PRKCH	18	14	28
PRKCI	PRKCI	37	0	40
PRKCQ	PRKCQ	10	32	18
PRKD1	PRKD1	0	0	0
PRKD2	PRKD2	0	0	0
PRKD3	PRKD3	12	46	35
PRKG1	PRKG1	8	4	9
PRKG2	PRKG2	34	0	20
PRKR	EIF2AK2	0	0	0
PRKX	PRKX	0	24	8
PRP4	PRPF4B	33	0	34
PYK2	PTK2B	0	0	6
QSK	KIAA0999	0	0	17
RAF1	RAF1	33	7	22
RET	RET	0	0	0
RET(M918T)	RET	0	0	0
RET(V804L)	RET	8	0	0

RET(V804M)	RET	1	5	1
RIOK1	RIOK1	39	0	13
RIOK2	RIOK2	0	26	48
RIOK3	RIOK3	20	16	23
RIPK1	RIPK1	0	0	0
RIPK2	RIPK2	47	56	56
RIPK4	RIPK4	18	4	34
RIPK5	DSTYK	30	8	72
ROCK1	ROCK1	0	0	0
ROCK2	ROCK2	0	0	0
ROS1	ROS1	3	0	31
RPS6KA4(Kin.Dom.1-N-terminal)	RPS6KA4	2	0	6
RPS6KA4(Kin.Dom.2-C-terminal)	RPS6KA4	0	0	0
RPS6KA5(Kin.Dom.1-N-terminal)	RPS6KA5	0	16	12
RPS6KA5(Kin.Dom.2-C-terminal)	RPS6KA5	0	0	0
RSK1(Kin.Dom.1-N-terminal)	RPS6KA1	15	17	4
RSK1(Kin.Dom.2-C-terminal)	RPS6KA1	0	4	0
RSK2(Kin.Dom.1-N-terminal)	RPS6KA3	48	45	65
RSK2(Kin.Dom.2-C-terminal)	RPS6KA3	3	0	0
RSK3(Kin.Dom.1-N-terminal)	RPS6KA2	10	7	33
RSK3(Kin.Dom.2-C-terminal)	RPS6KA2	28	6	19
RSK4(Kin.Dom.1-N-terminal)	RPS6KA6	47	67	63
RSK4(Kin.Dom.2-C-terminal)	RPS6KA6	24	20	40
S6K1	RPS6KB1	31	0	24
SBK1	SBK1	19	18	20
SGK	SGK1	0	0	0
SgK110	SgK110	18	18	6
SGK2	SGK2	7	12	22
SGK3	SGK3	0	0	0
SIK	SIK1	14	0	0
SIK2	SIK2	10	7	18
SLK	SLK	8	0	54
SNARK	NUAK2	0	0	10
SNRK	SNRK	38	0	0
SRC	SRC	0	1	11
SRMS	SRMS	70	14	62
SRPK1	SRPK1	76	81	43
SRPK2	SRPK2	0	0	11
SRPK3	SRPK3	0	7	0
STK16	STK16	24	3	0
STK33	STK33	30	37	45
STK35	STK35	0	0	12
STK36	STK36	22	3	14
STK39	STK39	11	0	15
SYK	SYK	0	0	0
TAK1	MAP3K7	0	0	46
TAOK1	TAOK1	53	0	61
TAOK2	TAOK2	82	0	62
TAOK3	TAOK3	15	0	37
TBK1	TBK1	36	32	66
TEC	TEC	16	12	0
TESK1	TESK1	34	16	46
TGFBR1	TGFBR1	0	5	18
TGFBR2	TGFBR2	0	0	0
TIE1	TIE1	22	17	35
TIE2	TEK	18	3	0
TLK1	TLK1	19	18	23
TLK2	TLK2	38	24	39
TNIK	TNIK	26	23	30
TNK1	TNK1	14	0	23
TNK2	TNK2	0	2	18
TNNI3K	TNNI3K	0	17	0

TRKA	NTRK1	1	3	5
TRKB	NTRK2	0	9	20
TRKC	NTRK3	0	0	22
TRPM6	TRPM6	0	3	9
TSSK1B	TSSK1B	0	0	0
TTK	TTK	36	0	0
TXK	TXK	42	40	68
TYK2(JH1domain-catalytic)	TYK2	21	0	22
TYK2(JH2domain-pseudokinase)	TYK2	0	0	0
TYRO3	TYRO3	10	5	15
ULK1	ULK1	0	4	42
ULK2	ULK2	0	0	0
ULK3	ULK3	0	5	20
VEGFR2	KDR	0	0	0
VRK2	VRK2	0	43	94
WEE1	WEE1	0	23	5
WEE2	WEE2	0	3	0
WNK1	WNK1	0	0	26
WNK3	WNK3	0	0	0
YANK1	STK32A	18	0	0
YANK2	STK32B	0	0	33
YANK3	STK32C	0	6	17
YES	YES1	0	0	23
YSK1	STK25	0	0	0
YSK4	YSK4	0	50	93
ZAK	ZAK	33	36	63
ZAP70	ZAP70	0	0	0
	**Selectivity (# kinases inh >50%):**	**19**	**20**	**48**
