# Supplementary material for: PERK Is a Haploinsufficient Tumor Suppressor: Gene Dose Determines Tumor-Suppressive Versus Tumor Promoting Properties of PERK in Melanoma
Source: PLoS Genet. 2016 Dec 15;12(12):e1006518. doi: 10.1371/journal.pgen.1006518 (PMC5207760; doi:10.1371/journal.pgen.1006518)
Supplement: S3 Table — (PDF) [file pgen.1006518.s010.pdf]

S3 Table. p53 mutations detected in the Perk+/- mouse melanomas

**p53 mutations**
S149A
T155S
M246I
R248Q
R282W
